# Solid Multiresponsive Materials Based on Nitrospiropyran-Doped Ionogels

**DOI:** 10.1021/acsami.1c04159

**Published:** 2021-05-31

**Authors:** Sara Santiago, Pablo Giménez-Gómez, Xavier Muñoz-Berbel, Jordi Hernando, Gonzalo Guirado

**Affiliations:** † Departament de Química, Universitat Autònoma de Barcelona, Bellaterra, Barcelona 08193, Spain; ‡ Instituto de Microelectrónica de Barcelona (IMB-CNM, CSIC), Bellaterra, Barcelona 08193, Spain

**Keywords:** ionogel, molecular switches, spiropyrans, smart materials, ionic liquids, smart devices

## Abstract

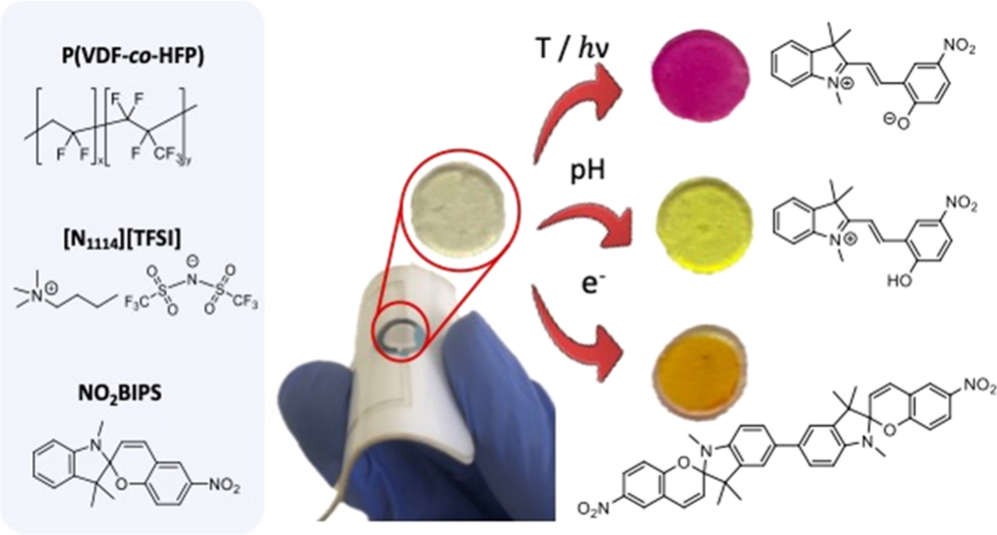

The application of molecular switches for the fabrication of multistimuli-responsive chromic materials and devices still remains a challenge because of the restrictions imposed by the supporting solid matrices where these compounds must be incorporated: they often critically affect the chromic response as well as limit the type and nature of external stimuli that can be applied. In this work, we propose the use of ionogels to overcome these constraints, as they provide a soft, fluidic, transparent, thermally stable, and ionic-conductive environment where molecular switches preserve their solution-like properties and can be exposed to a number of different stimuli. By exploiting this strategy, we herein pioneer the preparation of nitrospiropyran-based materials using a single solid platform that exhibit optimal photo-, halo-, thermo-, and electrochromic switching behaviors.

## Introduction

Smart functional solid materials that exhibit multistimuli-responsive behavior are of crucial importance for the construction of novel dynamic systems and devices.^1−5^ A major toolbox toward this goal are molecular switches.^6−11^ Among them, spiropyrans are frequently preferred due to their capacity to reversibly interconvert between states with strikingly different properties (e.g., color and polarity) upon application of a broad range of stimuli.^12−15^ On the one hand, spiropyran switches are well known to photoisomerize between their colorless spirocyclic (**SP**) and colored merocyanine isomers (**MC**).^15−17^ On the other hand, they have also been found to respond to other external stimuli^18−20^ such as pH,^21−23^ metal ions,^24−27^ solvent polarity,^28−30^ and redox potentials.^31−33^ In some cases, this allows the formation of other states apart from **SP** and **MC** (e.g., the protonated merocyanine state **MCH**^**+**^, the spiropyran dimer **SP**–**SP**) that additionally modify the photochromic response of the system,^21,23,25−27,30^ which further enriches the stimulus-sensitive activity of spiropyran switches.

Despite their broad functionality and versatility, the application of spiropyrans (and other switches) to the fabrication of truly smart materials and devices suffers from a major bottleneck: the influence of the surrounding matrix when these compounds are transferred from solution to a solid state (the so-called matrix effect), which often dramatically alters their switching performance.^34−37^ Two main factors account for this behavior: (a) the large geometrical changes needed to interconvert between the spirocyclic and open states of spiropyrans, which are hindered in rigid environments; and (b) the strong interaction that takes place with the surrounding solid matrix, which may vary the relative energy of their different states. Although these matrix effects could be exploited to develop new stimuli-sensitive responses for spiropyrans,^28,34,38^ they eventually prevent direct transfer of the optimal switching properties found in solution to the final materials. One of the main strategies proposed to overcome this drawback comprises properly selecting the nature of the matrix as to warrant minimal interaction with the switch and/or provide it with sufficient free volume as to fairly preserve its solution-like stimulus-sensitive response.^37^ This is the case of nanoporous solids (e.g., metal–organic^39,40^ and covalent organic^41^ frameworks) and soft polymeric matrices (e.g., low-*T*_g_ polymeric domains,^36,42,43^ polymer gels^44−46^).

**Scheme 1:**
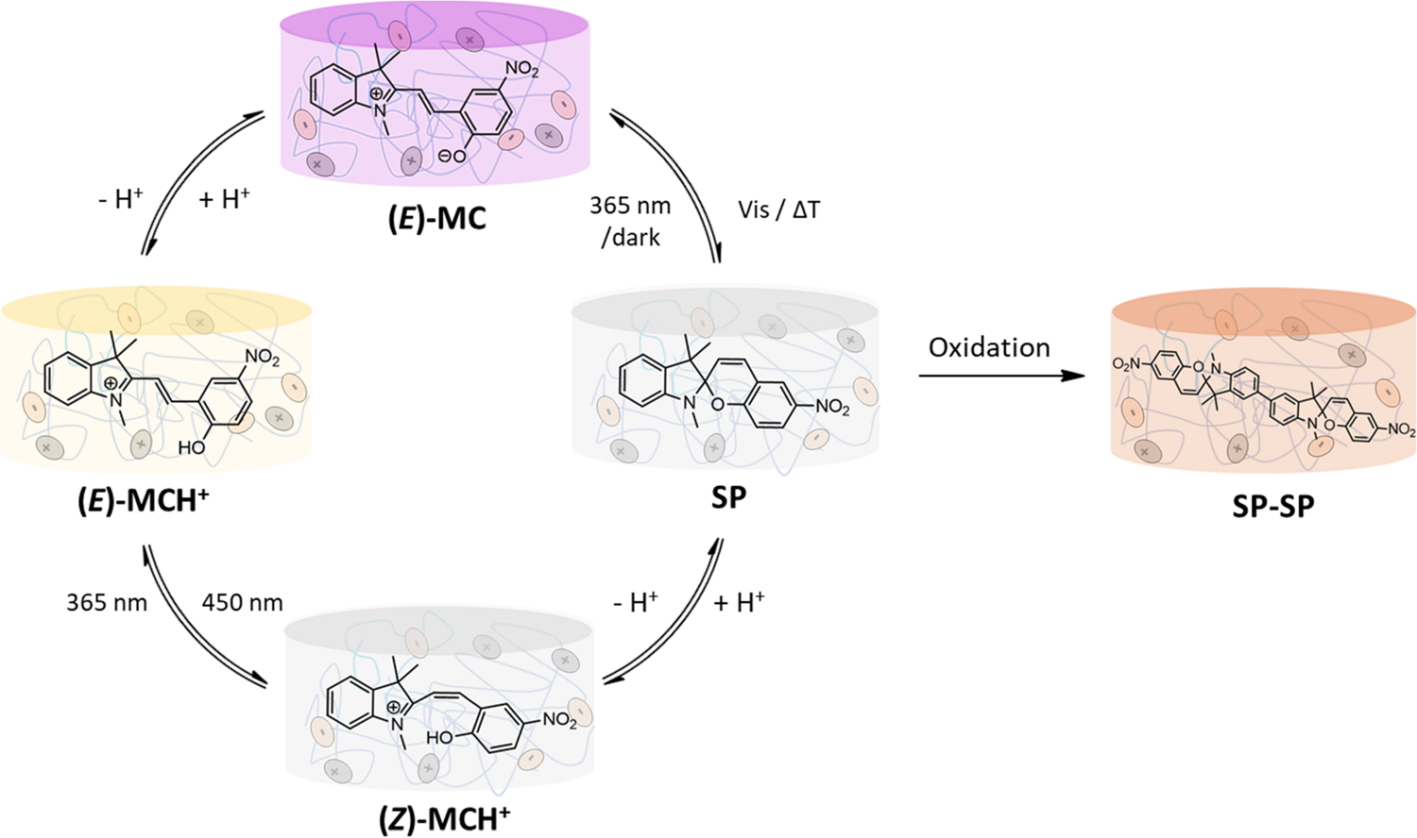
Multistimuli-Responsive Spiropyran-Based Ionogel Membranes (NO_2_BIPS@IG) to Be Prepared in This Study, Which Respond to Different Types of Input Signals: Light, Temperature, pH, and Electrical Current

However, even if a suitable spiropyran–matrix combination is chosen to reach optimal switching, the number and type of stimuli that can be applied to the resulting material are ultimately limited by the properties of the matrix, i.e., opaque materials will restrict spiropyran photochromism to the surface layer, whereas electroinduced responses could only be obtained for conductive substrates. In fact, the latter most probably justifies why the electrochromic response of spiropyrans has only been explored to date in solution with a proper supporting electrolyte and organic solvents.^31−33^ Therefore, to fully unleash the potential of the multistimuli-responsive behavior of spiropyrans, the development of versatile platforms that allow both solution-like switching and multiple operations under different input signals (e.g., light, electricity, pH, temperature, ions) is required. To reach this goal, we propose, herein, the use of ionogels (IGs), solid-gel polymer electrolytes that are attracting increasing attention for the fabrication of functional devices because of their unique combination of properties (i.e., elasticity, flexibility, easy preparation methodology, transparency, high ionic conductivities, and large electrochemical and chemical stability).^47−50^ Although a very recent example of spiropyran-based IG has been reported, only its light-sensitive operation has been described so far.^51^ Accordingly, in this work, we aim to demonstrate for the first time the multi-addressability of this class of materials, which would open the door for the fabrication of spiropyran-based smart devices with a broad variety of applications such as optical memories, electrochemical sensors, biosensors, and molecular actuators. For this, we focused our attention on 1′,3′-dihydro-1′,3′,3′-trimethyl-6-nitrospiro[2*H*]-1-benzopyran-2,2′-(2*H*)-indole (NO_2_BIPS) as a benchmark system, a well-known commercial nitrospiropyran derivative capable of responding to a plethora of external stimuli (Scheme 1).^21,31,33,52−54^

## Results and Discussion

### Fabrication of Spiropyran-Based Ionogel Membranes

**Figure 1:**
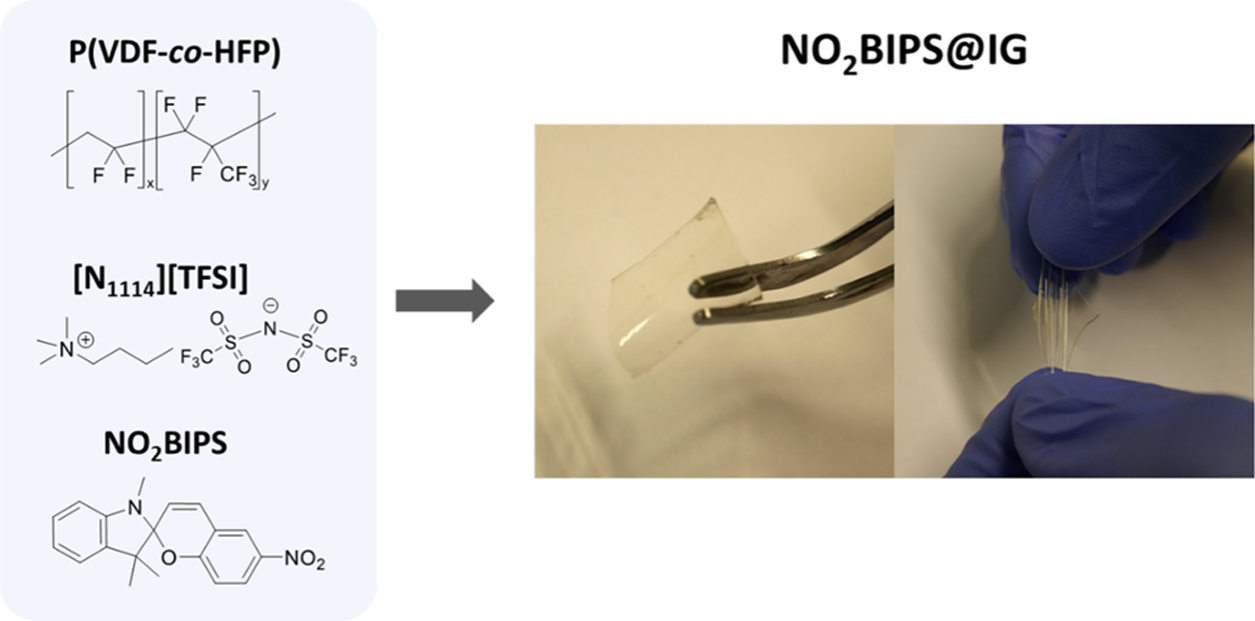
Key components for the fabrication of multistimuli-responsive NO_2_BIPS@IG films, which were found to be transparent, flexible, and elastic.

Based on our previous experience on the preparation of ionogels loaded with molecular switches,^55^ IG membranes were prepared by blending a fluorinated polymer (poly(vinylidene fluoride-*co*-hexafluoropropylene), P(VDF-*co*-HFP)), the trimethylbutylammonium bis(trifluoromethylsulfonyl)imide ionic liquid ([N_1114_][TFSI]), and NO_2_BIPS in acetone. After solvent evaporation, rubbery IG films containing free NO_2_BIPS molecules were obtained (NO_2_BIPS@IG, Figure 1), which were found to be transparent, flexible, and stretchable. These features arise from the convenient choice of the polymer network and the ionic liquid, where [N_1114_][TFSI] acts as a plasticizer favoring segmental mobility of the polymer chains and, hence, accounting for the final mechanical properties of the mixture. In addition, the ionic liquid provides free carriers to the material and explains the large ionic conductivities measured for the IG films (0.3 mS cm^–1^). As for the spiropyran content in NO_2_BIPS@IG, it was selected to meet two important criteria: (a) high color contrast upon application of different stimuli that could be properly quantified by means of ultraviolet–visible (UV–vis) absorption spectroscopy, and (b) good solubility in the liquid phase of the ionogel to enable optimal switching performance (*c* = 0.05–0.5 mg NO_2_BIPS/g IG). Actually, the formation of microscopic aggregates of the spiropyran molecules in this work was not observed for any of the concentrations tested when inspecting the ionogels under the optical microscope, which contributes to their high optical transparency.

Other advantages arise from our fabrication method of spiropyran-based IGs. On the one hand, it is very simple and, in contrast to previous reports,^44,45,51^ it can be directly applied to commercially available switches such as NO_2_BIPS without the need of further derivatization to warrant functionalization of the liquid or solid phases of the gel. Instead, the spiropyran molecules just lie dissolved in the ionic liquid phase of our ionogels, which provides them with a soft, fluidic and conductive environment to facilitate NO_2_BIPS switching upon illumination, addition of a chemical agent (e.g., acid), or application of an electrical current. In spite of this, it must be noted that no leakage of NO_2_BIPS molecules from NO_2_BIPS@IG films was observed in our experiments even when they were put in contact with external solutions (Figure S1). On the other hand, owing to the mechanical strength and the self-standing character of the IGs prepared, they can be easily shaped by cutting, thus offering significant advantages in the design and fabrication of smart devices using different printing methods (e.g., screen-printing or inkjet). Overall, our methodology for the preparation of spiropyran-doped IGs as versatile switching platforms drops the fabrication costs and complexity while granting access to the manufacture of flexible and stretchable smart devices.

### Photochromism of NO_2_BIPS@IG Membranes

**Figure 2:**
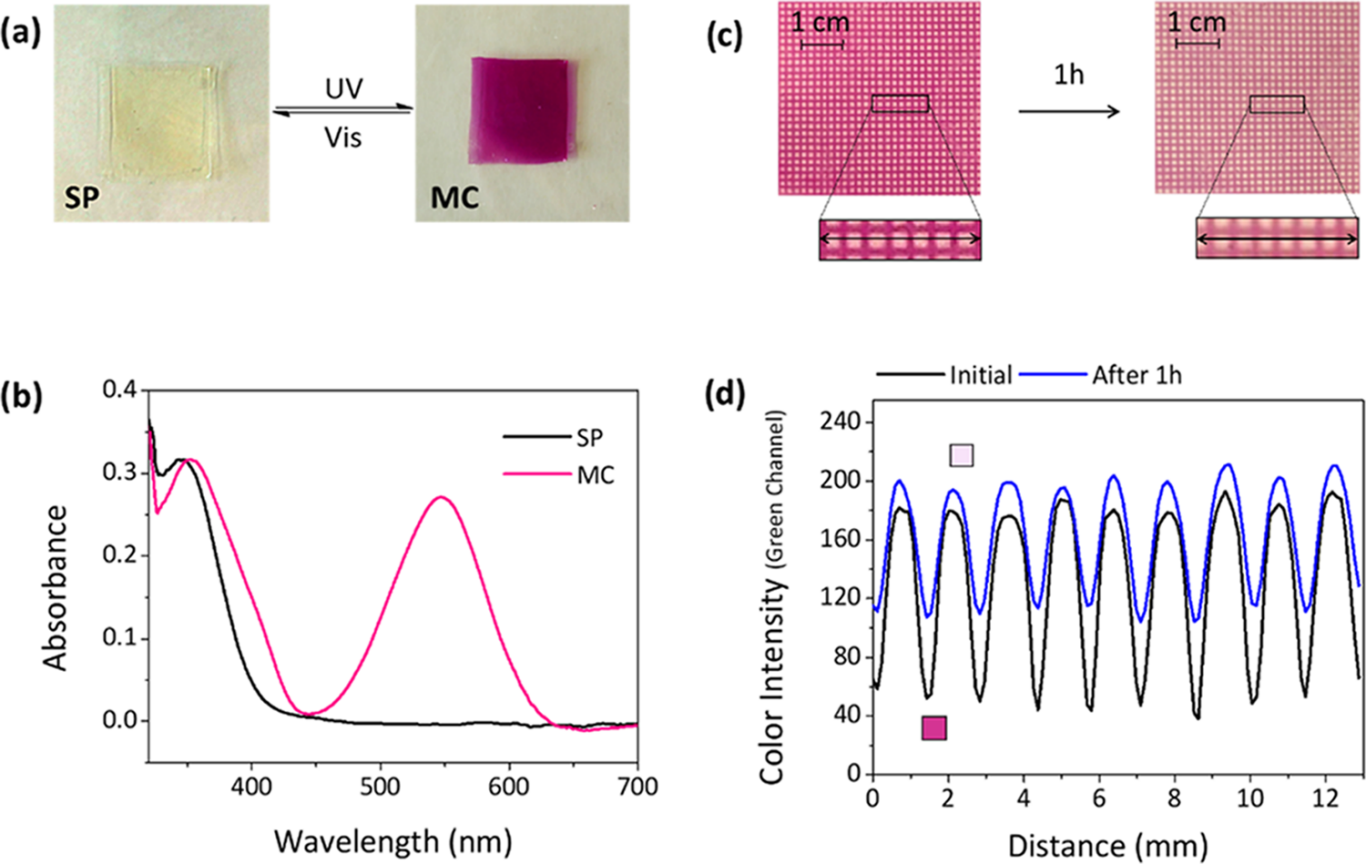
(a) Photochromic behavior of NO_2_BIPS@IG membranes when irradiated with UV and visible light to interconvert between the **SP** and **MC** states (*c*_NO_2_BIPS_ = 0.05 mg NO_2_BIPS/g IG). (b) UV–vis absorbance spectra of the **SP** (initial) and **MC** states (λ_exc_ = 365 nm until the photostationary state (PSS) is achieved) of NO_2_BIPS@IG membranes. (c) Optical images of a NO_2_BIPS@IG membrane exposed to UV light (λ_exc_ = 365 nm) through a photoprotective patterned mask, after the PSS is reached (left) and after 1 h under dim UV irradiation (right) to minimize the thermal back-isomerization process. The black rectangle indicates the region of the membrane that was analyzed. (d) Color intensity cross section of the images in (c) for the membrane region within the rectangle.

The photochromic interconversion between **SP** and **MC** states still remains the most exploited switching mechanism for spiropyrans,^11^ and it has been widely studied in solution for NO_2_BIPS.^54,56^ Therefore, it must be accurately preserved in the ionogels prepared for these materials to be of relevance. As observed in solution, the most stable isomer of NO_2_BIPS found in NO_2_BIPS@IG was the **SP** form, which mainly absorbs in the UV region (λ_abs_ = 346 nm) and makes the ionogel films essentially colorless and transparent at naked eye (Figure 2a,b). Actually, the UV–vis absorption spectrum measured for NO_2_BIPS@IG fairly reproduced the behavior in the [N_1114_][TFSI] solution and other aprotic polar solvents (Figure S2), thus indicating that the spiropyran molecules are mainly solvated by the ionic liquid in the ionogel.

After UV irradiation (λ_exc_ = 365 nm), intense purple coloration of NO_2_BIPS@IG was observed, which is indicative of extensive photoisomerization to the ring-opening isomer **MC** of the switch (Figure 2a).^54,56^ In particular, a new absorption band in the visible region characteristic of **MC** formation was found (λ_abs_ = 548 nm, Figure 2b), which preserves the same spectral features registered in acetonitrile and [N_1114_][TFSI] solutions (Figure S2). This is a clear proof that polar **MC** molecules also lie well dissolved in the liquid phase of the ionogels at the concentrations studied in this work, as significant spectral changes should have occurred in the case of aggregation.^57^ To assess the efficiency of the photocoloration process in the IG films, two different parameters were evaluated and compared to the behavior of NO_2_BIPS in solution. First, the total conversion from **SP** to **MC** was found to be around 22% for the photostationary state (PSS) generated in NO_2_BIPS@IG upon irradiation at 365 nm, a value rather similar to that measured for [N_1114_][TFSI] solutions (28%). Second, the quantum yield of the ring-opening photoisomerization process was calculated to be Φ_**SP**-**MC**_ = 0.15 ± 0.03 for NO_2_BIPS@IG membranes, which is in good agreement with the behavior reported for NO_2_BIPS in polar aprotic solvents (Φ_**SP**-**MC**_ = 0.24 and 0.12 in acetone and acetonitrile, respectively).^54^ Therefore, no detrimental effects on **SP**-to-**MC** photoisomerization were observed upon introduction of NO_2_BIPS molecules in ionogel films.

As for the reverse back-isomerization process, it was investigated both thermally and photochemically for NO_2_BIPS@IG. On the one hand, we observed that **MC**-to-**SP** back-isomerization in the dark followed a first-order kinetics with a rate constant of *k*_**SP**-**MC**_ = 9.2 × 10^–4^ s^–1^ at room temperature (Figure S3). This value is rather similar to those measured in the [N_1114_][TFSI] solution (1.90 × 10^–3^ s^–1^, Figure S3) and in solvents of high polarity (*k*_**SP**-**MC**_ = 1.0 × 10^–3^ s^–1^ in ethanol at 25 °C^58^), which further confirms that NO_2_BIPS molecules lie nonaggregated in solution-like domains within the ionogel films where their intrinsic photochromic properties are preserved. In addition, because of the high polarity of the ionic liquid phase of the ionogel that favors stabilization of the **MC** form, NO_2_BIPS@IG shows a rather slow thermal decoloration process, which might be exploited for the preparation of long-lived printed patterns on the ionogels (Figure 2c,d). This is favored by the restricted diffusion mobility of spiropyran molecules within the membranes, which is much slower than that in liquid solution. As a result, embedding NO_2_BIPS inside the ionogel matrix allows spatial confinement of the photoisomerized molecules within the irradiated areas for rather long periods (Figure 2c,d). On the other hand, if color fading is to be accelerated, irradiation with visible light can be exploited to induce fast **MC**-to-**SP** photoisomerization, which we found to occur in NO_2_BIPS@IG at similar rates as in the [N_1114_][TFSI] solution. Actually, this allowed conducting repetitive SP–MC photoconversion cycles by sequential illumination with UV and visible light, which demonstrate the reversible and robust photoresponse of NO_2_BIPS in the ionogels prepared (Figure S4).

### Photohalochromism and Thermochromism of NO_2_BIPS@IG Membranes

When dissolved in the ionic liquid [N_1114_][TFSI], NO_2_BIPS preserves the photohalochromic behavior already described in other polar solvents such as acetonitrile (Figure S5), which is attributed to the basicity of the 4-nitrophenolate group of its open form.^21^ Thus, upon addition of a strong acid (e.g., HClO_4_) in the dark, the spirocyclic structure of the **SP** isomer opens to yield the (*Z*)-**MCH**^**+**^ species, where the exocyclic carbon–carbon double bond maintains the cis configuration of the initial compound and its phenolate moiety is protonated (Scheme 1). This process, which can be reverted by the addition of a base, leads to a new absorption band at λ_abs_ = 303 nm characteristic of the (*Z*)-**MCH**^**+**^ form. As a consequence, the solution remains essentially colorless. Similarly, acid–base titration of a solution of the **MC** isomer results in reversible formation of its protonated state (*E*)-**MCH**^**+**^ with trans configuration and λ_abs_ = 392 nm, which makes the system turn from purple to yellow color (Scheme 1). In addition, the (*Z*)-**MCH**^**+**^ and (*E*)-**MCH**^**+**^ forms preserve the photochromic properties of the nonprotonated SP–MC couple, and they can be reversibly interconverted upon carbon–carbon double bond photoisomerization with UV and violet-blue light, respectively (Scheme 1).

**Figure 3:**
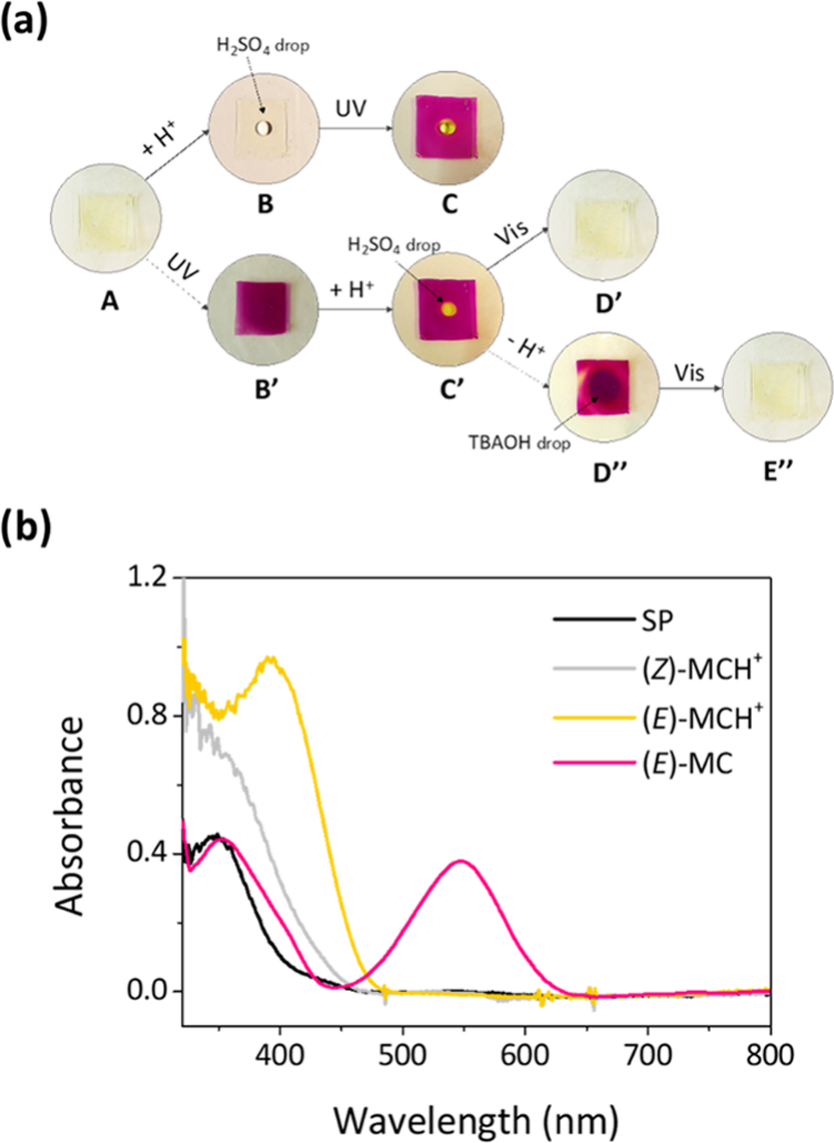
(a) Optical response of NO_2_BIPS@IG (*c*_NO_2_BIPS_ = 0.05 mg NO_2_BIPS/g IG) upon light and pH changes. (A) initial color (**SP** form), (B) drop of 10 mM H_2_SO_4_ solution, (C) irradiation at λ_exc_ = 365 nm. (B′, C′) UV irradiation of A (λ_exc_ = 365 nm) followed by addition of H_2_SO_4_, (D′) irradiation of C′ at λ_exc_ = 445 nm, (D″, E″) addition of tetrabutylammonium hydroxide (TBAOH 10 mM) followed by visible light exposure (λ_exc_ = 445 nm). (b) UV–vis absorption spectra of the initial state of NO_2_BIPS@IG (**SP**) and upon acidification ((*Z*)-**MCH**^**+**^), and UV irradiation of (*Z*)-**MCH**^**+**^ ((*E*)-**MCH**^**+**^) and **SP** (**MC**).

Interestingly, when embedded in IG membranes, NO_2_BIPS molecules show a very similar photohalochromic behavior, probably due to the fact that they are principally solvated by the ionic liquid (Figure 3a,b). In particular, no change in color was observed when a droplet of diluted H_2_SO_4_ was placed on top of NO_2_BIPS@IG, though an increase in the absorption at λ_abs_ < 400 nm was registered, which is compatible with (*Z*)-**MCH**^**+**^ formation. Unfortunately, competitive absorption by the ionogel matrix prevented proper determination of the absorption spectral maximum of this species at λ_abs_ ∼310 nm. In spite of this, (*Z*)-**MCH**^**+**^ generation could be corroborated by subsequent UV irradiation (λ_exc_ = 365 nm). While most of the membrane turned purple colored because of **SP**-to-**MC** photoisomerization, the area in contact with the acid droplet became yellow colored as expected for the (*Z*)-**MCH**^**+**^-to-(*E*)-**MCH**^**+**^ photoconversion process. The same effect was observed when the chemical and optical stimuli were applied in inverse order, which proves the capacity of the spiropyran molecules within the ionogel to undergo **MC**-to-(*E*)-**MCH**^**+**^ transformation. Furthermore, all of these processes could be reverted by illumination with visible light and/or addition of a base, thus eventually recovering the initial colorless and transparent state of NO_2_BIPS@IG. Therefore, our results demonstrate the potential of spiropyran-loaded IGs for the preparation of photohalochromic solid materials, as they allow the properties of the embedded switches to be preserved, warrant the access of wet chemicals (i.e., acid and base solutions) to the matrix, and enable confinement of the halochromic behavior to the regions of the system that are in contact with those chemicals. However, it must be mentioned that prolonged acid–base treatment of the IG membranes affected its chemical stability, which we attribute to the base-induced Hofmann elimination reaction of the quaternary ammonium cation of the [N_1114_][TFSI] IL.^58^ As a consequence, a limited number of halochromic and photohalochromic cycles could be conducted before observing the degradation of the material (Figure S6).

**Figure 4:**
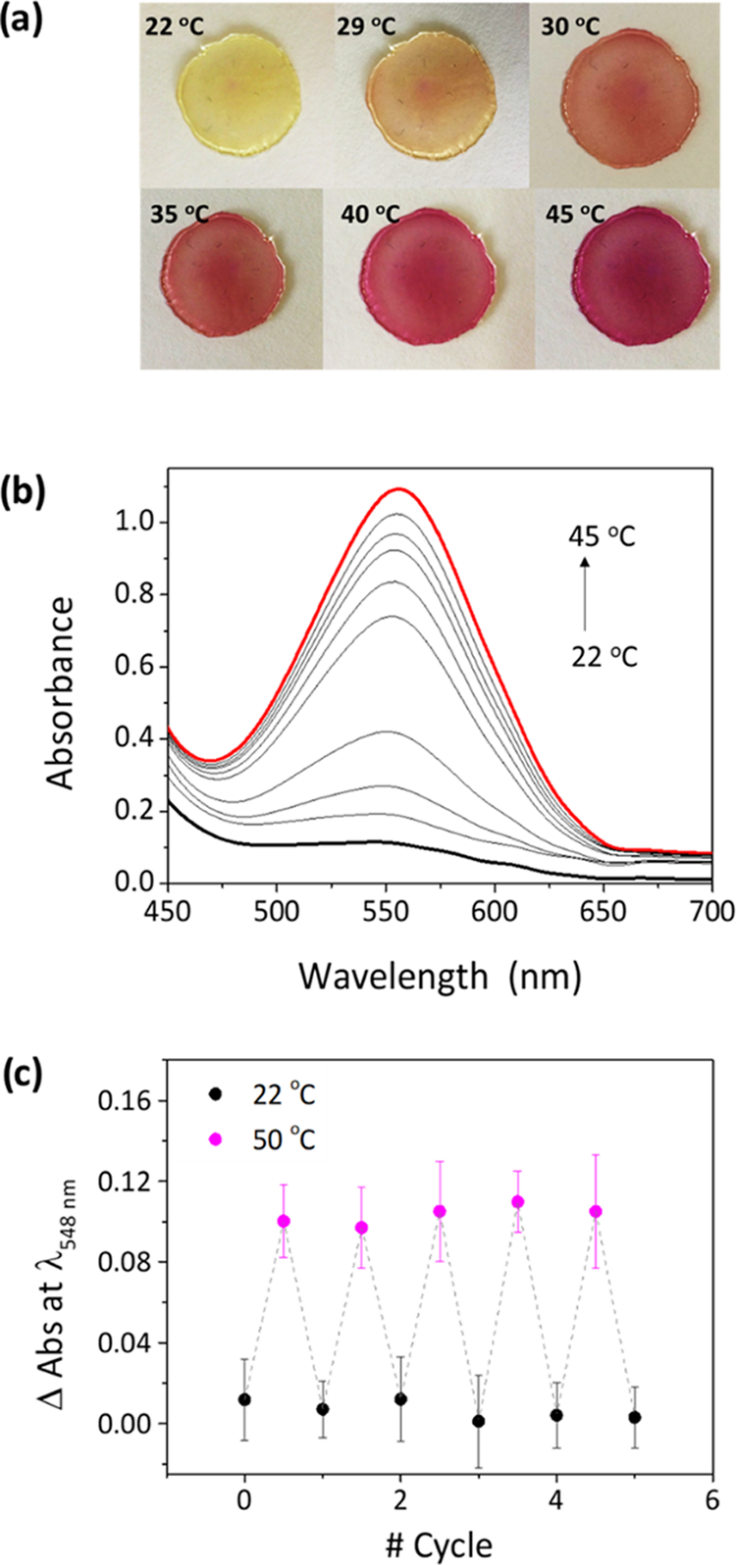
(a) Color change of NO_2_BIPS@IG membranes (*c*_NO_2_BIPS_ = 0.5 mg NO_2_BIPS/g IG) when increasing the temperature gradually. (b) Variation of the UV–vis absorbance spectrum of NO_2_BIPS@IG with temperature. (c) Variation of the absorbance at the spectral maximum of the **MC** isomer in NO_2_BIPS@IG upon five consecutive thermochromic cycles. Average data is shown for three independent measurements in different membranes.

Another stimulus to which spiropyrans can respond is temperature, as thermal heating can induce heterolytic cleavage of the C–O_spiro_ bond of **SP** to yield the corresponding **MC** isomer. Although this process is not typically favored in organic media where **SP** is the most stable isomer, it could eventually occur if two main conditions are fulfilled: (a) the presence of electron-withdrawing groups stabilizing the negative charge of the phenolate moiety of **MC**, as it is the case of the nitro substituent in NO_2_BIPS; and (b) dissolution in highly polar media that further contributes to the stabilization of the zwitterionic **MC** isomer (e.g., in water–methanol mixtures).^59^ In view of this, the thermochromic behavior might be exhibited by NO_2_BIPS@IG when the spiropyran molecules lie solvated by the highly polar ionic liquid [N_1114_][TFSI]. Indeed, clear coloration both in the [N_1114_][TFSI] solution (Figure S7) and in the ionogel films was observed by just heating above 30 °C, and maximum **MC** absorption was registered at 45 °C for NO_2_BIPS@IG that did not further increase at higher temperatures (λ_abs_ = 552 nm, Figure 4a,b). From the UV–vis absorption data, the maximum thermal isomerization yields in the [N_1114_][TFSI] solution and in the membranes were estimated to be 4 and 5%, respectively. This demonstrates that the thermochromic conversion from **SP** to **MC** is less efficient than that achieved upon exposure to light, probably due to the insufficient stabilization of the merocyanine form by the surrounding ionic liquid; however, the color change induced was clear and vivid enough as to be easily seen with naked eye.

It must be noted that a minor spectral shift was measured in our thermochromic experiments relative to the absorption of photochemically generated **MC** molecules (λ_abs_ = 548 nm). Although this must be ascribed to a simple thermal effect on absorption, it cannot be overlooked that the thermal ring-opening of the **SP** form of NO_2_BIPS has been reported to yield different stereoisomers of **MC** bearing distinct optical properties instead of just the predominant (*E*)-**MC** structure obtained upon photoisomerization. Independent of this, fast decoloration of NO_2_BIPS@IG was measured after subsequently cooling the ionogels down to room temperature, which demonstrates the reversibility of the thermochromic behavior. This was possible owing to the exceptional stability of the IGs prepared even at high temperatures, which results from the negligible vapor pressure and intrinsic thermal stability of ionic liquids (ILs). Indeed, multiple thermochromic cycles could be measured for NO_2_BIPS@IG membranes without apparent degradation (Figure 4c). Therefore, these results pave the way for the fabrication of low-cost thermochromic materials based on spiropyrans for smart labeling and packaging.

### Electrochromism of NO_2_BIPS@IG Membranes

**Figure 5:**
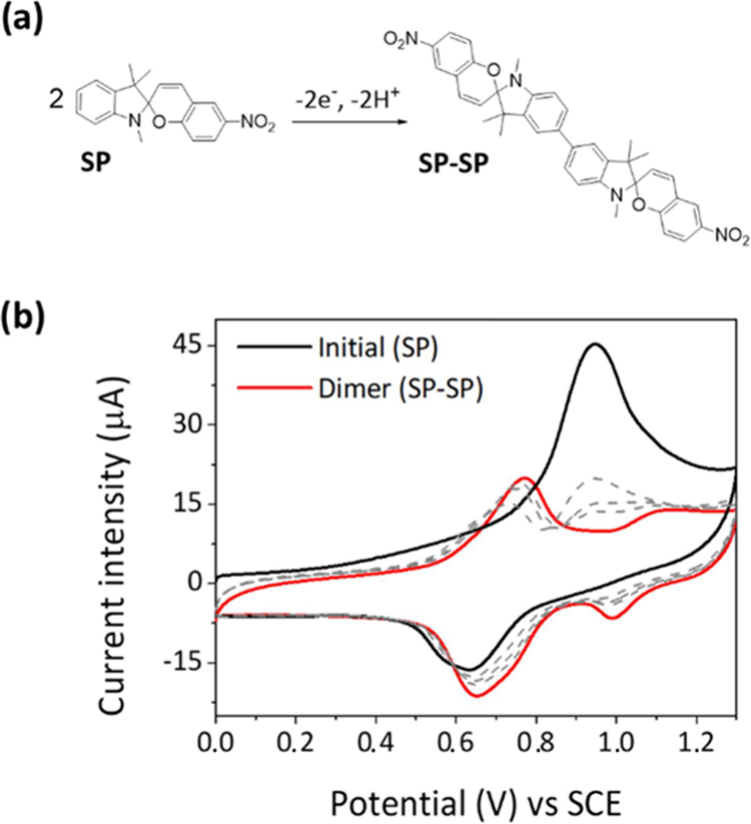
(a) Electrochemical conversion of NO_2_BIPS into the dimeric species **SP**–**SP** upon oxidation.^33,60,61^ (b) Cyclic voltammograms of NO_2_BIPS@IG (*c*_NO_2_BIPS_ = 0.5 mg NO_2_BIPS/g IG) registered for a freshly prepared ionogel (solid black line) and after 30 consecutive oxidation cycles (red solid line, scan rate: 20 mV s^–1^). Gray dashed lines correspond to intermediate voltammograms measured.

In contrast to their photochromic, photohalochromic, and thermochromic behaviors, the electrochromic properties of spiropyrans have been less exploited, probably because of the difficulty to achieve redox-induced switching in solid materials. It is, therefore, in this area where the ionogels prepared in this work are expected to have a greater impact, as their large conductivities should enable the electrochemical operation of spiropyran switches. In light of this, the electrochromic and electrochemical properties of NO_2_BIPS@IG were thoroughly investigated, for which we built on the previous findings about the redox-induced behavior of NO_2_BIPS in solution: it dimerizes upon oxidation (Figure 5a).^33,60,61^

Based on that, we focused on analyzing the response of NO_2_BIPS@IG upon electrochemical oxidation. Figure 5b shows the anodic region of the cyclic voltammogram of NO_2_BIPS@IG, which presents a one-electron and irreversible oxidation wave at +0.95 V (vs Ag/AgCl) associated with the oxidation of the amino group of the indoline moiety to the corresponding radical cation. After 30 consecutive anodic cycles at 50 mV s^–1^, this wave disappeared, while two new oxidation signals at +0.77 and +1.10 V (vs Ag/AgCl) emerged with half the intensity. These observations are in good agreement with previous electrochemical results in solution,^32,33,60,61^ and they are indicative of a dimerization process of NO_2_BIPS to yield an **SP**–**SP** dimer via oxidative carbon–carbon bond formation. In fact, the oxidation waves at +0.77 and +1.10 V (vs Ag/AgCl) are attributed to the formation of the radical cation and dication of the dimer, respectively, while two new signals are also observed in the cathodic region of the voltammogram that correspond to the sequential reduction of the two nitro groups of the dimer (*E*_red_ = −0.8 and −1.1 V (vs Ag/AgCl), Figure S8). Therefore, these results prove that the electrochemical behavior of NO_2_BIPS@IG in solution can be directly transferred to the solid state by means of ionogel matrices. Actually, **SP** electrodimerization in these materials might be even favored by two additional factors. First, since the diffusion of NO_2_BIPS molecules is largely restricted in the IL phase of the gel, the reactivity between the nearby **SP** radical cations must be further promoted. In fact, this effect has already been observed upon immobilization of NO_2_BIPS onto surfaces, which assisted the electrochemical oxidative C–C aryl coupling of the switch. Second, the use of ionic liquids in IGs should also increase the stability of the reactive radical cation species through solvation, thus aiding the dimerization reaction.^62^

**Figure 6:**
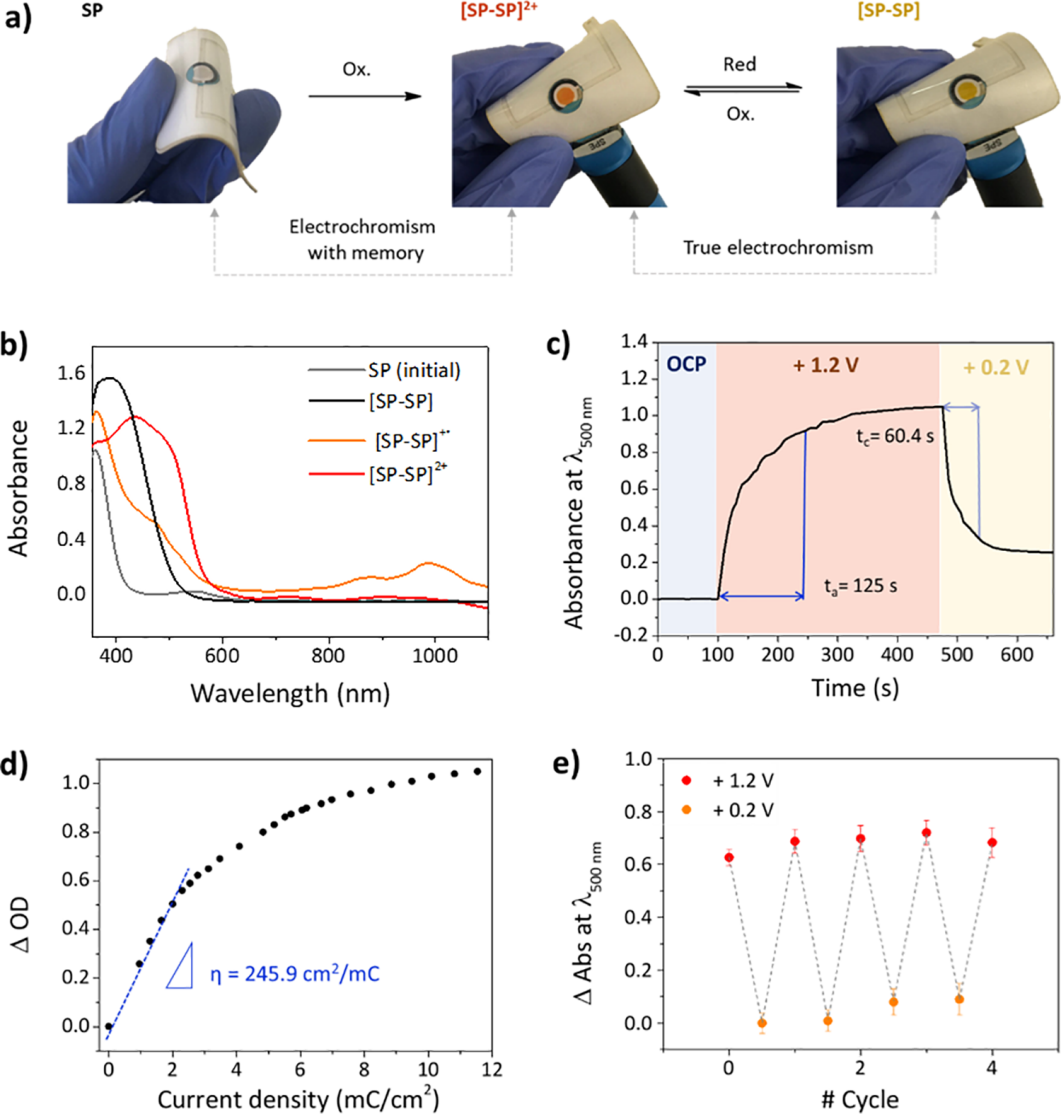
(a) Flexible display with a three-electrode compartment. The NO_2_BIPS@IG membrane (*c*_NO_2_BIPS_ = 0.5 mg NO_2_BIPS/g IG) is in contact with an ITO-SPE working electrode (WE) and changes its color when applying electric potentials to induce irreversible **SP**-to-**[SP–SP]**^**2+**^ and reversible **[SP–SP]**^**2+**^-**[SP–SP]** transformations. (b) UV–vis absorption spectra of the initial state of NO_2_BIPS@IG (**SP**) and of the different redox products formed: [**SP**–**SP]**, [**SP**–**SP**]^·**+**^, and **[SP–SP]**^**2+**^. (c) Change in absorbance monitored at λ_abs_ = 500 nm for the electrochromic device shown in (a) before applying any electric potential (OCP, 0–100 s), at *E*_ap_ = +1.2 V (vs Ag/AgCl, 100–475 s), and at *E*_ap_ = +0.2 V (vs Ag/AgCl, 475–655 s). (d) Plot of the optical density difference at λ_abs_ = 500 nm against the current density passed at *E*_ap_ = +1.2 V (vs Ag/AgCl) for the electrochromic device shown in (a). (e) Switching color reversibility measured at λ_abs_ = 500 nm for the same electrochromic device after applying consecutive cycles of *E*_ap_ = +1.2 V (vs Ag/AgCl) for 130 s and *E*_ap_ = +0.2 V (vs Ag/AgCl) for 70 s. Average data is shown for three independent measurements in different membranes.

It is important to highlight that when applying +1.2 V (vs Ag/AgCl) using either a carbon screen-printed electrode (SPE) or an ITO-SPE electrode as a working electrode (WE), the electrochemical formation of the dimer in NO_2_BIPS@IG is accompanied by a pronounced change in the color of the material, which turns intensely reddish-orange. Two main factors account for this behavior. First, **SP** dimerization is immediately followed by oxidation to the dicationic state of the dimer [**SP**–**SP**]^2+^ at the applied potential, thus leading to the overall redox-induced **SP**-to-[**SP**–**SP**]^2+^ transformation (Figure 6a). Second, the UV–vis absorption spectrum of the dicationic dimer [**SP**–**SP**]^2+^ is bathochromically shifted with respect to the **SP** species (λ_abs_ = 416 and 500 nm for [**SP**–**SP**]^2+^; Figure 6b) and, therefore, the ionogel becomes colored.

As **SP** electrodimerization is irreversible,^33,60,61^ the coloration effect observed in NO_2_BIPS@IG at *E*_ap_ = +1.2 V (vs Ag/AgCl) could not be reverted and a permanent modification of the initial colorless membrane was provoked, i.e., the material presents “electrochromism with memory” under these conditions (Figure 6a). Spectroelectrochemical measurements were conducted to characterize this electrochromic behavior, for which we fabricated a flexible device with a built-in three-electrode electrochemical cell where a 4 mm in diameter circular NO_2_BIPS@IG membrane was deposited onto an ITO-SPE working electrode (Figures 6a and S10). On the one hand, a high color contrast was found in this process, as proven by the large change in transmittance measured at λ_abs_ = 500 nm when transforming **SP** into [**SP**–**SP**]^2+^ (Δ*T*_1_ = 90%, Figure S9). On the other hand, the switching time needed to produce 90% of such color change was observed to be rather long (*t*_a_ = 125 s, Figure 6c), probably because the electrochromic conversion implies a dimerization reaction that is limited by the spatial encounter between two **SP** molecules within the ionogel matrix where diffusion is restricted. Finally, the electrochromic efficiency (η) of the **SP**-to-**[SP–SP]**^**2+**^ transformation was extracted from the slope of the linear region of the plot between the change in the optical density (ΔOD) and the current density needed to produce this change (Figure 6d). The obtained value, η = 245.9 cm^2^ mC^–1^, is somewhat lower than other reported results for electrochromic devices,^63^ due to the large capacitive current of both the NO_2_BIPS@IG membrane and the screen-printed ITO electrode used.

The electrochromic behavior of NO_2_BIPS@IG can be further expanded by taking advantage of the reversible reduction of [**SP**–**SP**]^2+^ to its radical cation [**SP**–**SP**]^·+^ and its neutral form [**SP**–**SP**] (e.g., at *E*_ap_ = +0.2 V (vs Ag/AgCl)). These species show new different absorption bands in the visible (λ_abs_ = 360 and 473 nm for [**SP**–**SP**]^·+^ and λ_abs_ = 385 nm for [**SP**–**SP**]; Figure 6b) and near-IR regions (λ_abs_ = 874 and 984 nm for [**SP**–**SP**]; Figure 6b), some of which might be related to the intervalence charge transfer between the monocation and dication species.^64^ As a consequence, a clear color change from reddish-orange to yellow takes place upon [**SP**–**SP**]^2+^-to-[**SP**–**SP**] transformation, which can be reverted after subsequent oxidation (Figure 6a). Therefore, this opens the door to use previously oxidized NO_2_BIPS@IG membranes as “true electrochromic” systems, a behavior that we also characterized by means of spectroelectrochemical measurements on the flexible device shown in Figure 6a. In this case, the color contrast for the reversible electroswitching between [**SP**–**SP**]^2+^ and [**SP**–**SP**] was associated with a transmittance change at λ_abs_ = 500 nm of Δ*T*_2_ = 47% (Figure S9), while the response time determined was significantly faster (*t*_c_ = 60.4 s, Figure 6c). As for the electrochromic reversibility and fatigue resistance of the [**SP**–**SP]**^**2+**^-[**SP**–**SP**] system, it was investigated upon consecutive applied voltages of *E*_ap_ = +1.2 and +0.2 V (vs Ag/AgCl) for 130 and 70 s, respectively (Figure 6e). After five oxidation–reduction cycles, the electrochromic response of the membranes remained rather stable as proven by the similar absorbance values measured at λ_abs_ = 500 nm for the dication and neutral forms of the spiropyran dimer. These results, together with the flexibility of the electrochemical device prepared, make NO_2_BIPS@IG membranes very appealing electrochromic materials for the fabrication of systems of great technological interest, such as wearable sensors^65^ and flexible panels.^66^

### Multistimuli-Responsive Displays Based on NO_2_BIPS@IG

**Figure 7:**
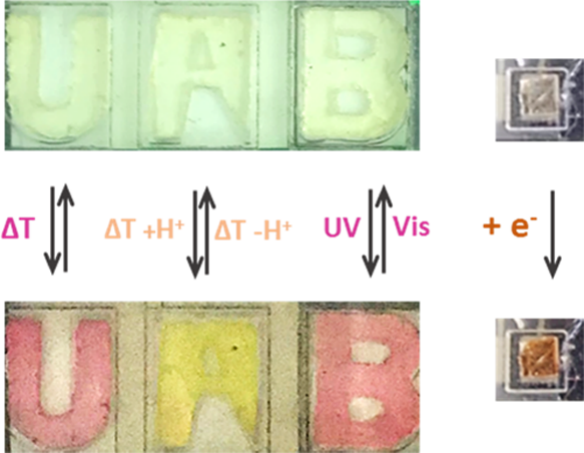
Multistimuli-responsive microfluidic prototype device based on NO_2_BIPS@IG shaped into letters and a square (*c*_NO_2_BIPS_ = 0.5 mg NO_2_BIPS/g IG). Each form was exposed to different external stimuli: *T*, pH, light, and electric potential.

To demonstrate the feasibility of spiropyran-loaded ionogels for practical applications, we built a simple microfluidic architecture with multistimuli-responsive performance (Figure S11). In this device, we introduced four different NO_2_BIPS@IG membranes cut with distinct complex shapes using a CO_2_ ablation laser, for which we took advantage of their high mechanical strength and high thermal and chemical stability. Each one of those membranes could then be exposed independently to stimuli of variable nature, as shown in Figure 7. For the U-shaped membrane, a hot liquid flow (*T* = 40 °C) was passed through the microfluidic cell to induce the thermochromic conversion into a pink-colored **MC** isomer. The A-shaped membrane was instead put into contact with a hot acidic solution (*T* = 40 °C), which led to the thermohalochromic formation of the yellow-colored (*E*)-**MCH**^**+**^ form. In the case of the B-shaped membrane, the pink-colored **MC** state was reached upon UV irradiation (λ_exc_ = 365 nm) through a near-UV and visible transparent window. Finally, to trigger the electrochromic response of the system, the square-shaped NO_2_BISP@IG membrane was placed on top of a platinum (Pt) electrode and an irreversible color change from transparent to orange was obtained because of **SP** electrodimerization to yield [**SP**–**SP**]^2+^ at *E*_ap_ = +1.0 V (vs Pt). Hence, selective and reversible thermo-, halo-, photo-, and electrochromic responses could be measured for each one of them by appropriately selecting the stimulus of interest.

**Figure 8:**
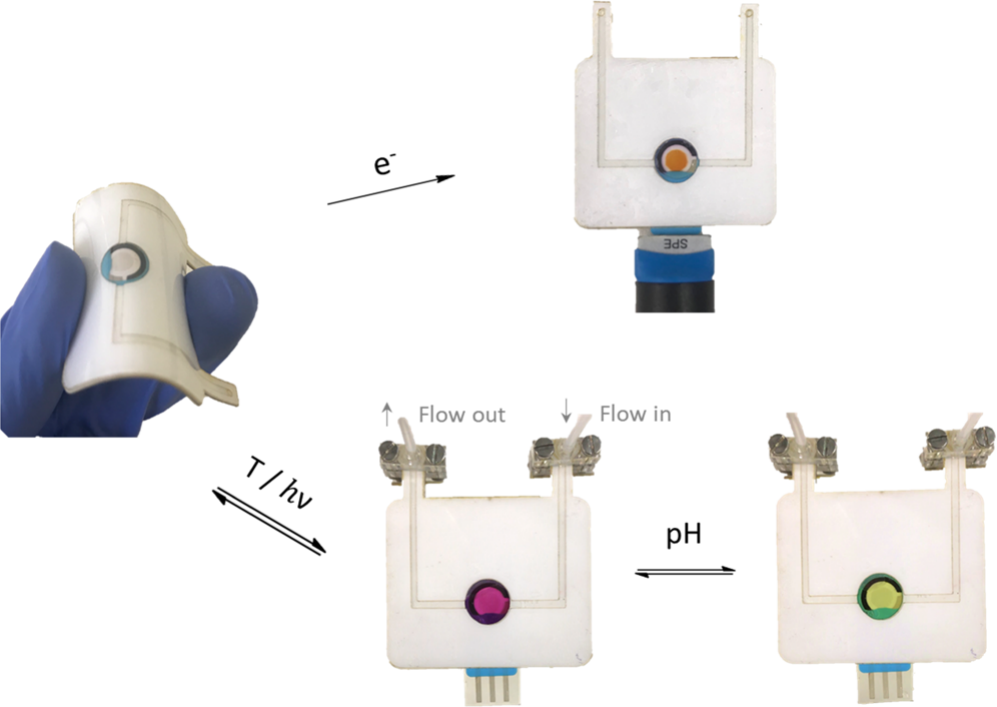
Flexible multistimuli-responsive microfluidic prototype based on NO_2_BIPS@IG (*c*_NO_2_BIPS_ = 0.5 mg NO_2_BIPS/g IG). The material shows different color responses upon application of distinct external stimuli in a single device: *T*, pH, light, and electrical potential.

To further demonstrate the potential of spiropyran-loaded ionogels for the fabrication of smart devices, we decided to exploit their good mechanical properties for the preparation of multiresponsive flexible displays. With this aim, a microfluidic channel was incorporated to the flexible electrochromic device shown in Figure 6a, thus enabling the application of other stimuli different from electrical potentials to NO_2_BISP@IG (Figures 8 and S10). Thus, the introduction of hot water and aqueous acidic solutions resulted in color changes compatible with the transformation of the colorless initial **SP** molecules in the ionogel to the pink **MC** isomer and the yellow (*E*)*-***MCH**^**+**^ protonated species. In addition, **SP**-to-**MC** conversion could also be induced under UV irradiation, while the system preserved its capacity to turn reddish-orange when applying a potential of +1.2 V (vs Ag/AgCl) to induce **[SP–SP]**^**2+**^ formation. These results, together with those shown for the rigid microfluidic prototype, are unambiguous proofs that ionogels offer a wide range of advantageous properties (i.e., facile formulation, high ionic conductivities and optical transparency, chemical and thermal stability, access to chemicals from external solutions, and mechanical strength and flexibility) that make them a very promising platform for the fabrication of smart devices based on multistimuli-responsive molecular switches.

## Conclusions

In this work, we have demonstrated the potential of ionogels to be used as platforms for the preparation of smart materials based on multistimuli-responsive molecular switches. With this aim, we formulated ionogels from a polyfluorinated polymer and an ionic liquid that were loaded with the photo-, halo-, thermo-, and electrosensitive NO_2_BIPS spiropyran derivative. The advantages of the resulting NO_2_BIPS@IG membranes were found to be multifold. First, the solution-like photochromic properties of the switch were preserved in the ionogels thanks to the light transparency of these materials and the fluid nature of their ionic liquid phase where NO_2_BIPS molecules lie, which favors the large conformational changes needed for the switching process to occur. The latter also grants fast access of chemicals to NO_2_BIPS@IG (e.g., acids and bases), which we exploited to trigger the characteristic halo- and photohalochromic operations of the embedded switch molecules. In addition, because of the nonvolatility of ionic liquids, the ionogel membranes obtained could be heated without degradation to stimulate the thermochromic behavior of NO_2_BIPS. More importantly, NO_2_BIPS@IG strongly benefits from the adequate ionic conductivity provided by the ionic liquid phase of the material, which enabled the electroinduced operation of the molecular switch. All of these features, in combination with the facile preparation, flexibility, mechanical strength, self-standing nature, and shapeability of ionogels, make these materials promising candidates for the fabrication of a range of stimuli-sensitive systems and devices for optoelectronic applications (e.g., smart displays, chemical sensors, security inks, data storage). As a proof of concept, we constructed rigid and flexible microfluidic prototype devices containing different NO_2_BIPS@IG membranes that could be independently exposed to external stimuli to selectively promote their photo, halo-, thermo-, and electrochromic response.

## Experimental Section

### Materials

1′,3′-Dihydro-1′,3′,3′-trimethyl-6-nitrospiro[2*H*-1-benzopyran-2,2′-(2*H*)-indole] (NO_2_BIPS) was purchased from TCI Chemicals (≥98% pur.) and used without further purification. Poly(vinylidene fluoride-*co*-hexafluoropropylene) (P(VDF-*co*-HFP)) and HCl 36.5–38% were acquired from Merck and used as received. Trimethylbutylammonium bis(trifluoromethylsulfonyl)amide ([N_1114_][TFSI]) was purchased from Solvionic and dried under vacuum using molecular sieves for 24 h to ensure a total water content lower than 0.001%. Acetone was purchased from Acros (≥99.5% pur.) and used as provided.

### Preparation of NO_2_BIPS@IG Membranes

For NO_2_BIPS@IG preparation, P(VDF-*co*-HFP) and the ionic liquid [N_1114_][TFSI] were mixed in acetone in a 1:5 weight ratio. This mixture was stirred overnight at room temperature and under a N_2_ atmosphere until the polymer was fully dissolved, and it was finally sonicated for 3 min. Later, the desired amount of NO_2_BIPS was added to the solution and dissolved by stirring. The concentration of NO_2_BIPS in the ionogel membranes (NO_2_BIPS@IG) was selected taking into account the film thickness (typically, ∼60 μm) to obtain absorbance values below 1 in the UV–vis spectra. In most of the cases, this was observed for NO_2_BIPS contents in the range of *c*_NO_2_BIPS_ = 0.05–0.5 mg NO_2_BIPS/g IG. The resulting viscous solution was cast into a ceramic evaporating dish and was left at room temperature for 24 h until the solvent was completely evaporated. Eventually, a transparent, flexible, and elastic thin film was obtained. Films could be stored for weeks in a glovebox without observing any detrimental effect in their stimuli-responsive properties.

### Characterization of NO_2_BIPS@IG Membranes

Ultraviolet–visible (UV–vis) absorption spectra were recorded in a Hamamatsu L10290 spectrophotometer and a HP 8453 spectrophotometer. Spectroelectrochemical studies were performed coupling a VSP100 potentiostat controlled by EC-Lab V9.51 software to the Hamamatsu L10290 spectrophotometer. Electrochemical and spectroelectrochemical measurements on NO_2_BIPS@IG membranes were performed using screen-printed electrodes (SPE, DropSens), a three-electrode system composed of a carbon or optically transparent ITO as a working electrode (WE), a carbon counter electrode (CE), and a Ag/AgCl reference electrode. Since NO_2_BIPS@IG membranes are photoresponsive, the spectroelectrochemical measurements were performed in discontinuous mode, recording the spectra in time for 0.5 s to avoid the long-time exposure of the sample to the light beam. An infrared probe (Laserliner ThermoSpot) was used for monitoring the temperature of the IG membrane when heated. To estimate the composition of the **SP**–**MC** mixtures prepared upon irradiation, the **MC** content in the resulting photostationary states was calculated by Lambert Beer’s equation using its absorption coefficient reported in ref (54). The **SP**-to-**MC** photoisomerization quantum yield in the ionogel membrane was determined using the methodology reported in refs (67) and (68), which is described in detail in the Supporting Information.^69^**SP**-to-**MC** photoisomerization was induced with a Vilber Lourmat UV lamp equipped with two 4 W tubes emitting at 365 nm or the third harmonic of a Nd:YAG ns-pulsed laser (Brilliant, Quantel, λ_exc_ = 365 nm), while **MC**-to-**SP** back-photoisomerization was triggered with a cw laser diode at λ_exc_ = 532 nm (Z-Laser). The thermal back-photoisomerization **MC**-to-**SP** process in solution and the membranes was investigated in the dark and at room temperature using the methodology described in the Supporting Information. For the photohalochromic study, an acidic 10 mM H_2_SO_4_ and a basic 10 mM TBAOH aqueous solutions were prepared, and a total volume of 20 μL was cast on the top of NO_2_BIPS@IG membranes. Depending on the state aimed to reach, a combination of an acidic or basic solution was used along with the irradiation at λ_exc_ = 445 nm (sciTec), λ_exc_ = 365 nm, (Brilliant, Quantel, λ_exc_ = 365 nm), or λ_exc_ = 532 nm (Z-Laser).

### Fabrication of Multistimuli-Responsive Devices

The portable rigid microfluidic system was designed and fabricated using low-cost polymers poly(methyl methacrylate) (PMMA), double-sided pressure-sensitive adhesive (PSA), and poly(dimethyl siloxane) (PDMS) to demonstrate the feasibility of NO_2_BIPS@IG in real scenarios (Figure S11). The polymers were fast-prototyped with a CO_2_-laser writer (Epilog Mini 24, Epilog Laser). The total size of the fluidic system was 9 mm in height, 43 mm in width, and 70 mm in length. The system was formed by two structures (bottom and top) of different layers of polymers. The bottom structure (Figure S11b) was formed by 2 PMMA layers bonded with a 175 μm thick PSA layer. A bottom black-colored 3 mm thick PMMA layer was used to avoid the light beam losses during light exposure of the NO_2_BIPS@IG material. The second 380 μm thick PMMA layer enabled the position of the chips used for the electrochromic tests. Two 11 × 9 mm^2^ silicon chips fabricated using standard photolithographic techniques were used in this system:^6^ a chip containing two in-parallel platinum (Pt) microelectrodes working as a working electrode (WE) and a chip containing three in-parallel Pt microelectrodes working as a counter (CE) and pseudo-reference electrodes (p-RE). The microelectrodes had an area of 2.5 or 5 mm^2^. The bottom structure is completed by a 680 μm PDMS layer defining the outline of the four NO_2_BIPS@IG material pieces used for the light, pH, temperature, and electric potential tests. This layer enabled the hosting of the NO_2_BIPS@IG pieces and their perfect alignment with the microfluidic cells defined in the top structure. Four shapes are defined for the tests: a letter U, a letter A, a letter B, and a square, which are used for the temperature, pH, light, and electric potential stimulus, respectively. Regarding the electrochemical cell, other shapes are defined in the PDMS to expose the microelectrodes used as CE and p-RE. Finally, two more rectangular shapes are defined to allow the electrical connection of the chip with the potentiostat equipment. The PDMS layer also disenabled the fluidic leakage between both structures during the fluidic tests. The top structure (Figure S11c) is formed by four 500 μm thick layers bonded by 175 μm thick PSA layers. These layers defined three 75 μL and one 37.5 μL microfluidic cells used for temperature, pH, light, and electric potential stimulus, respectively. The PMMA layers also enabled the fluidic connection between them, the position of the microfluidic threads used in each inlet and outlet for all cells, and the insertion of the two spring-loaded connectors (RS Components, Switzerland) used to contact the chips with the measurement instrument. Finally, both structures were fixed using screws (2 mm diameter) to allow easy assembly and disassembly of the system (Figure S11d). Samples were flowed inside the device using acidic diluted aqueous solution of HClO_4_ or basic diluted aqueous solution of TBAOH at different temperatures. However, in the case of the electrochemical compartment, no aqueous solution was flowed to avoid side reactions during the electrochromic performance. In this case, bare IG was used as a solid electrolyte to ensure adequate ionic conductivity.

The flexible multistimuli-responsive device (37.5 mm in width and 41 mm in length) was formed by five layers of fast-prototyped polymers mechanized with a laser writer (Figure S10). A 175 μm polycarbonate layer was used to close the microfluidic device and to define the position of the 1 mm in diameter microfluidic inlet and outlet (Layer 1). Layer 2 was made of a 175 μm thick PSA film, which defined the 1 mm in width microfluidic channels connecting the inlet and outlet in Layer 1 with a 20 μL electrochemical cell. Layer 3 was formed by a 175 μm thick PSA layer bonded to a 50 μm PMMA layer and was used to contact the electrochemical cell to the electrochemical sensor. The electrochemical sensor was fabricated with a Dropsens screen-printed ITO electrode and positioned using the hole defined in Layer 4 (175 μm thick double-sided PSA layer +50 μm PMMA layer). Finally, the flexible device was enclosed by a 50 μm thick PSA layer used as a white back-cover. A 4 mm in diameter circular NO_2_BIPS@IG membrane was deposited onto the ITO-SPE working electrode of the electrochemical sensor. Samples were flowed inside the device using an acidic diluted aqueous solution of HClO_4_ or a basic diluted aqueous solution of TBAOH at different temperatures.

## Supporting Information Available

NO_2_BIPS@IG membranes water stability; photoisomerization in acetonitrile and [N1114][TFSI] solutions; determination of photoisomerization quantum yields for NO_2_BIPS@IG membranes; thermal-back isomerization reaction in [N1114][TFSI] solution and the NO_2_BIPS@IG membrane; photoisomerization cycles in NO_2_BIPS@IG membrane; photohalochromism of NO_2_BIPS in acetonitrile and [N1114][TFSI]; halochromic cycles in NO_2_BIPS@IG membranes; thermochromism of NO_2_BIPS in [N1114][TFSI] solution; cyclic voltammetry of NO_2_BIPS@IG membranes; electrochromic characterization of NO_2_BIPS@IG membranes; fabrication of rigid microfluidic devices; fabrication of flexible devices (PDF)
