# Supplementary material for: Solid Multiresponsive Materials Based on Nitrospiropyran-Doped Ionogels
Source: ACS Appl Mater Interfaces. 2021 May 31;13(22):26461–71. doi: 10.1021/acsami.1c04159 (PMC8483435; doi:10.1021/acsami.1c04159)
Supplement: Supplementary file 1 — am1c04159_si_001.pdf [file am1c04159_si_001.pdf]

Supporting Information for:

SOLID MULTI-RESPONSIVE MATERIALS BASED ON NITROSPIROPYRAN-DOPED IONGELS

Sara Santiago[a],[b], Pablo Giménez-Gómez[b], Xavier Muñoz-Berbel[b], Jordi Hernando[a],*, and Gonzalo Guirado[a],*

[a] Departament de Química, Universitat Autònoma de Barcelona, Bellaterra (Barcelona), 08193, Spain.

[b] Instituto de Microelectrónica de Barcelona (IMB-CNM, CSIC), Bellaterra, (Barcelona), 08193, Spain.

E-mail: Jordi.Hernando@uab.cat; Gonzalo.Guirado@uab.cat

Table of Contents:

1. NO_2BIPS@IG membranes water stability ……………………………S3

2. Photoisomerization in acetonitrile and [N_{1114}][TFSI] solutions……....S4

3. Determination of photoisomerization quantum yields for NO_2BIPS@IG membranes ………………………………………………….……....S5

4. Thermal-back isomerization reaction in [N_{1114}][TFSI] solution and the NO_2BIPS@IG membrane……………….……………………………S7

5. Photoisomerization cycles in NO_2BIPS@IG membranes.....................S9

6. Photohalochromism of NO_2BIPS in acetonitrile and [N_{1114}][TFSI].......S7

7. Halochromic cycles in NO_2BIPS@IG membranes.............................S11

8. Thermochromism of NO_2BIPS in [N_{1114}][TFSI] solution……………....S12

9. Cyclic voltammetry of NO_2BIPS@IG membranes…………………......S13

10. Electrochromic characterization of NO_2BIPS@IG membranes…......S14

11. Fabrication of rigid microfluidic devices……...…………...……..S15

12. Fabrication of flexible devices…………………………..…………..S16

13. References…………………………………………………..…….S17

1. NO2BIPS@IG membranes water stability

NO_2BIPS@IG membranes containing the **SP** and **MC** forms of the switch were submerged in water for 1 h (Figure S1a.). After that, they were removed and dried at room temperature. Both the membranes and the water samples left were then separately irradiated at λ_{exc} = 365 nm. As shown, similar strong purple coloration was obtained for the two membranes, while no effect was observed for the incubation water samples. Hence, no signals in the UV and visible region arising from free NO_2BIPS molecules were observed for the water media that contained the membranes for 1 h (Figure S1b). This demonstrates that the IG membranes did not suffer any leakage of the loaded spiropyran switches when in contact with aqueous media.

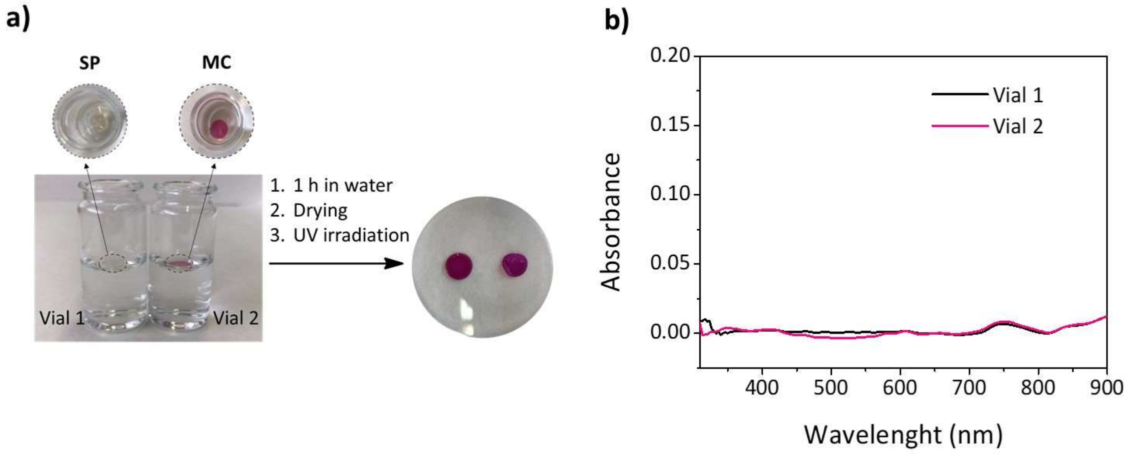

Figure S1. a) NO_2BIPS@IG membranes submerged in water for 1 h: dark-adapted (**SP**) and UV-irradiated (**MC**). **b)** UV-vis absorbance spectra of the water samples separated from the membranes and irradiated at λ_{exc} = 365 nm.

2. Photoisomerization in acetonitrile and [N_{1114}][TFSI] solutions

Thermally stable **SP** form photoisomerizes into **MC** after tens of seconds of irradiation until a photostationary state (PSS) is reached. From the absorption of the **MC** band at λ_{abs} ~ 550 nm and the accepted molar absorptivity of **MC** at its spectral maximum in polar solvents (ε_{MC} = 5.2 10^4 M^{-1} cm^{-1}),[1] the **MC** content in the PSS was estimated: 27 % in acetonitrile and 28% in N_{1114}TFSI solutions (Figure S2).

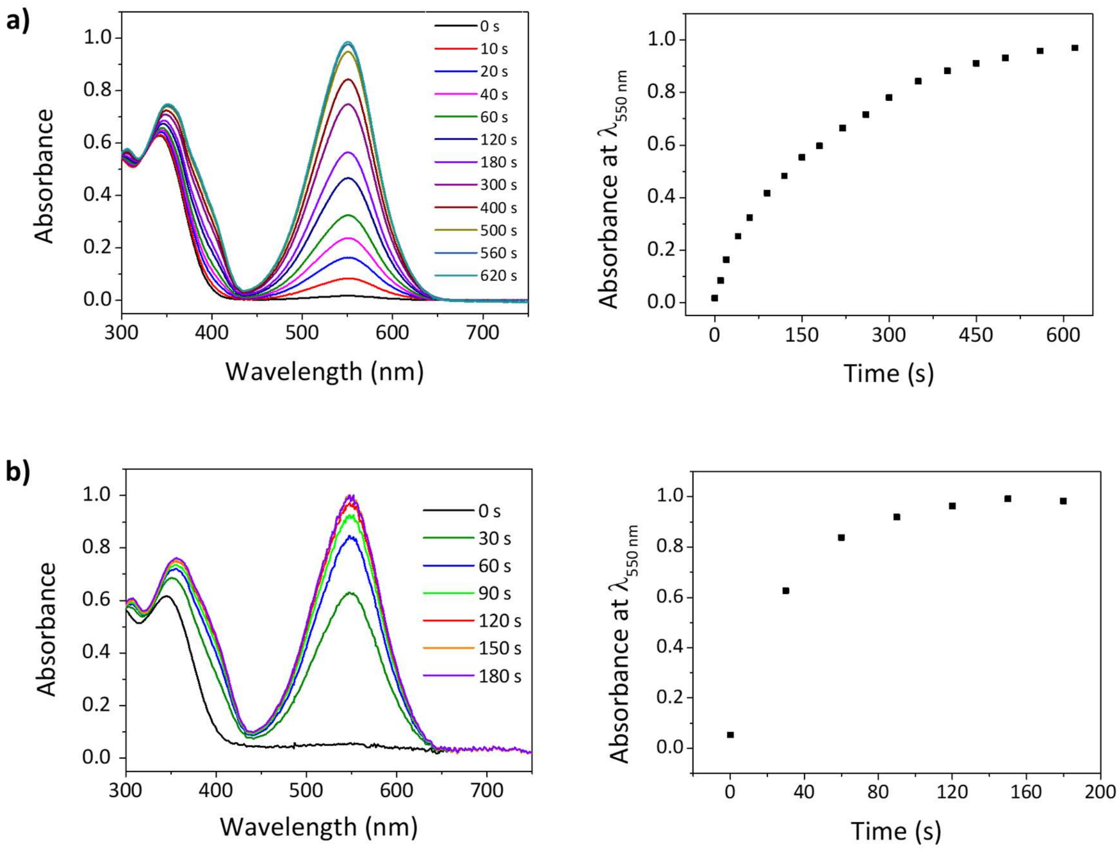

Figure S2. **a)** Variation of the UV-Vis absorbance spectra of a 0.5 mM NO_2BIPS solution in acetonitrile under irradiation at λ_{exc} = 365 nm. **b)** Variation of the UV-Vis absorbance spectra of a 0.5 mM NO_2BIPS solution in [N_{1114}][TFSI] under irradiation at λ_{exc} = 365 nm.

3. Determination of photoisomerization quantum yields for NO2BIPS@IG membranes

The **SP**-to-**MC** photoisomerization quantum yield in the ionogel membrane was determined using the methodology reported in refs. 1 and 2 and azobenzene as a reference compound ($\Phi_{trans\text{-}cis}$= 0.15 in acetonitrile[4]). In our photochromic system, the most stable **SP** form is converted upon irradiation at λ_{exc} = 365 nm into **MC**, which is short-lived and can photoisomerized back to **SP**. Therefore, the effect of both the photoinduced and thermal back-reactions should be taken into account. To do so, the following differential equation must be used to define the concentration of **SP** in time when $NO_2BIPS@IG$ membranes are continuously irradiated at λ_{exc}:

$$\frac{d[\mathbf{SP}]}{dt} = -\left[\left(\Phi_{SP\to MC}\,\varepsilon_{SP}^{\lambda exc} + \Phi_{MC\to SP}\,\varepsilon_{MC}^{\lambda exc}\right) I_0 \cdot l \cdot F + k_{MC\to SP}^{thermal}\right] \cdot [\mathbf{SP}]$$

$$+\left(\Phi_{MC\to SP}\,\varepsilon_{MC}^{\lambda exc} \cdot I_0 \cdot l \cdot F + k_{MC\to SP}^{thermal}\right) \cdot [\mathbf{SP}]_0 \qquad (1)$$

and

$$F = (1 - 10^{-Abs^{\lambda exc}})/Abs^{\lambda_{exc}} \qquad (2)$$

In these equations: F is the dimensionless photokinetic factor; $\Phi_{SP\to MC}$ and $\Phi_{MC\to SP}$ are the photoisomerization quantum yields of the forward and backwards photoreaction; $k_{MC\to SP}^{thermal}$ is the thermal back-isomerization rate constant, which was previously determined from UV-vis absorption measurements in the dark (see next section); $\varepsilon_{SP}^{\lambda exc}$ and $\varepsilon_{MC}^{\lambda ex}$ are the molar extinction coefficients of **SP** and **MC** at the excitation wavelength obtained from ref. [1]; $Abs^{\lambda_{exc}}$ is the total absorbance of the sample at the excitation wavelength; I_0 is the photon flux of the lamp, which was determined from the photoisomerization of the azobenzene reference; l is the optical path (i.e., the thickness of the IG); [**SP**] is the concentration of the **SP** form in the membranes at different irradiation times; and $[\mathbf{SP}]_0$ is the initial concentration of **SP** in the ionogel.

To determine $\Phi_{SP\to MC}$ and $\Phi_{MC\to SP}$, the variation in time of the absorbance at the spectral maximum of **MC** ($Abs^{\lambda_{abs}}$, λ_{abs} = 548 nm) was monitored at λ_{exc} = 355. In this case, $Abs^{\lambda_{abs}}$ follows the Lambert Beer law, where $\varepsilon_{SP}^{\lambda abs}$ is null at λ_{abs} = 548 nm:

$$Abs^{\lambda_{abs}} = [\left(\varepsilon_{SP}^{\lambda abs} - \varepsilon_{MC}^{\lambda abs}\right) \cdot [\mathbf{SP}] + \varepsilon_{MC}^{\lambda abs} \cdot [\mathbf{SP}]_0 \cdot l \qquad (3)$$

From these measurements, $\Phi_{SP \rightarrow MC}$ and $\Phi_{MC \rightarrow SP}$ were retrieved by fitting equation (3) to the experimental data, for which [**SP**] values were obtained in each iteration step by numerical integration of equation (1).

4. Thermal-back isomerization reaction in $[N_{1114}][TFSI]$ solution and the $NO_2BIPS@IG$ membrane

The rate constants of the thermal **MC**-to-**SP** back-isomerization reaction ($k_{MC\to SP}^{thermal}$) in solution and for $NO_2BIPS@IG$ membranes were determined by measuring the absorbance decay at λ_{abs} = 548 nm in time ($A^{\lambda_{548\,nm}}$) from the starting PSS mixture generated upon UV irradiation. As **MC**-to-**SP** thermal transformation follows a first order kinetics and **MC** is the only species absorbing at λ_{abs} = 548 nm, the absorbance decay measured must follow differential equation (4), which leads to the logarithmic dependence of $A^{\lambda_{548\,nm}}$ with time shown in equation (5).

$$-\frac{dA^{\lambda_{548\,nm}}}{dt} = k_{MC\to SP}^{thermal} A^{\lambda_{548\,nm}} \quad (4)$$

$$\ln A_t^{\lambda_{548\,nm}} = -k_{MC\to SP}^{thermal} \cdot t + \ln A_0^{\lambda_{548\,nm}} \quad (5)$$

Therefore, plotting the experimental values of $\ln(A_t^{\lambda_{548\,nm}}/A_0^{\lambda_{548\,nm}})$ against time gives a linear regression with slope equal to $-k_{MC\to SP}^{thermal}$ (Figure S3).

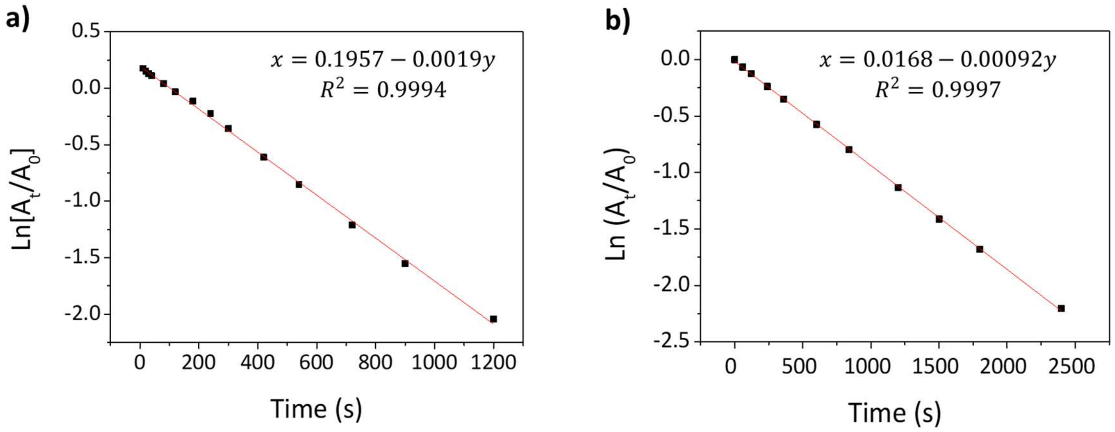

Figure S3. First order decay kinetics at room temperature corresponding to the thermal back-isomerization from **MC** to **SP** for: **a)** a 0.5 mM NO_2BIPS solution in $[N_{1114}][TFSI]$; **b)** a $NO_2BIPS@IG$ membrane.

5. Photoisomerization cycles in NO_2BIPS@IG membranes

The cycle stability of NO_2BIPS@IG membranes upon consecutive **SP-MC** photoisomerization processes was monitored by UV-vis absorption spectroscopy (Figure S4). No detrimental effects on the photochemical response of these materials were observed after 7 photoconversion cycles.

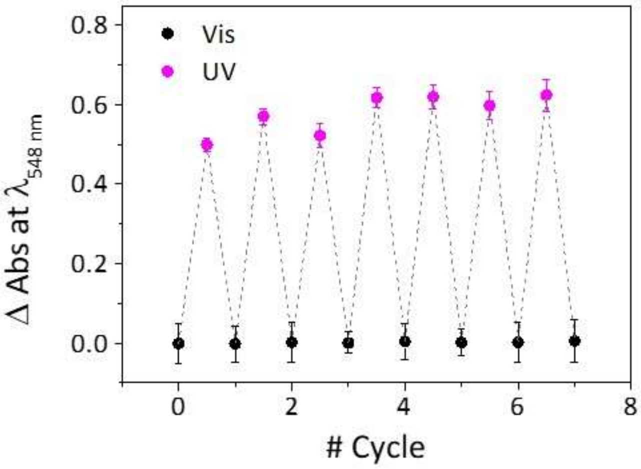

Figure S4. Variation of the absorbance at the spectral maximum of the **MC** isomer in NO_2BIPS@IG upon 7 consecutive cycles of illumination with UV (λ_{exc} = 365 nm, 80 s) and white light (80 s). Average data is shown for 3 independent measurements in different membranes.

6. Photohalochromism of NO_2BIPS in acetonitrile and $[N_{1114}][TFSI]$ solutions

Figures S5 (**a-b**) show the halochromic behavior of pure NO_2BIPS solutions in $[N_{1114}][TFSI]$ and acetonitrile monitored by UV-vis absorption spectroscopy. After addition of 1 equivalent of $HClO_4$, the corresponding protonated form (*Z*)-**MCH**$^+$ was generated, which was reverted back to **SP** by subsequent titration with 1 equivalent of tetrabutylammonium hydroxide (TBAOH). Figures S5 (**c-f**) show the combined photohalochromic behavior for the same solutions monitored by UV-vis absorption spectroscopy. In (**c**-**d**) the initial **SP** form was first converted into (*Z*)-**MCH**$^+$ after addition of 1 equivalent of $HClO_4$. Then, (*Z*)-**MCH**$^+$ was irradiated at λ_{exc} = 365 nm until reaching the PSS mixture (*Z*)-**MCH**$^+$:(*E*)-**MCH**$^+$, which was reverted back by illumination at λ_{exc} = 445 nm. In (**e-f**) the initial **SP** form was first converted into the PSS mixture **SP**:**MC** upon irradiation at λ_{exc} = 365 nm, which was then protonated with 1 equivalent of $HClO_4$ to produce the corresponding (*Z*)-**MCH**$^+$:(*E*)-**MCH**$^+$ mixture. Further addition of 1 equivalent of TBAOH and irradiation at λ_{exc} = 365 nm allowed retrieving the previous PSS mixture **SP**:**MC**.

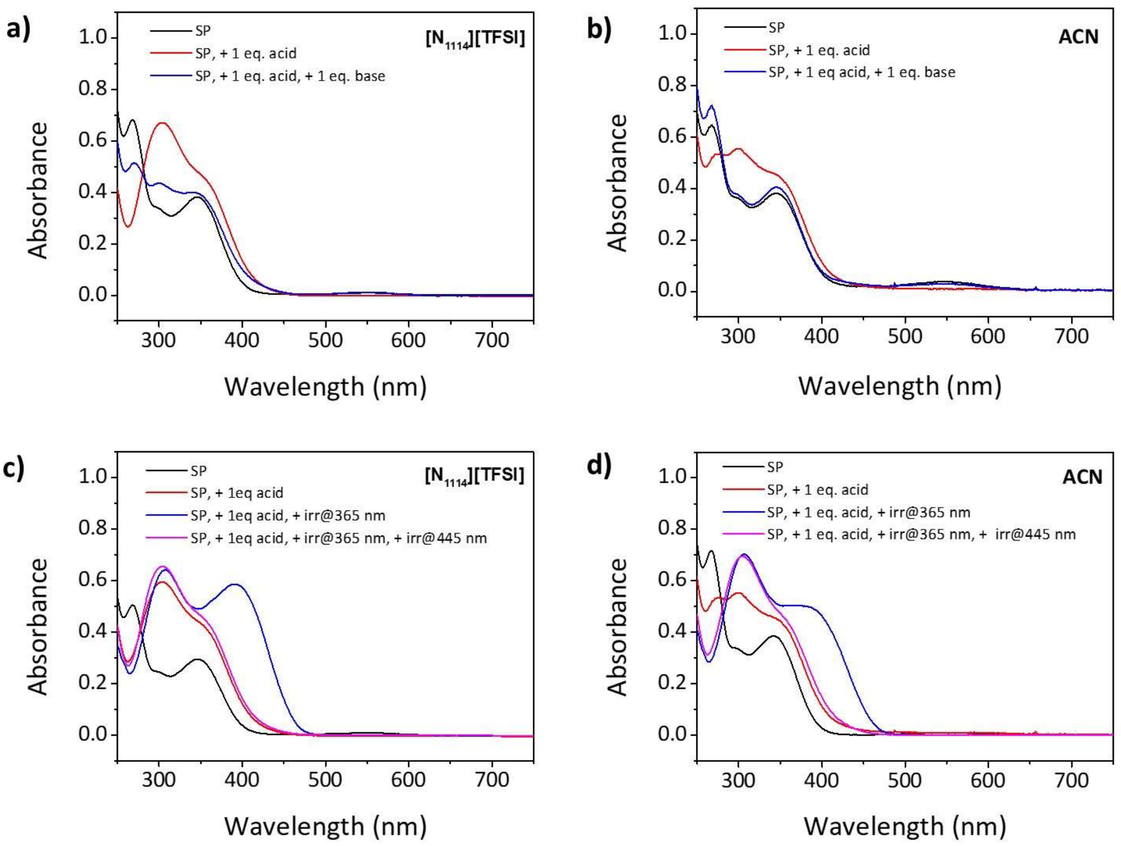

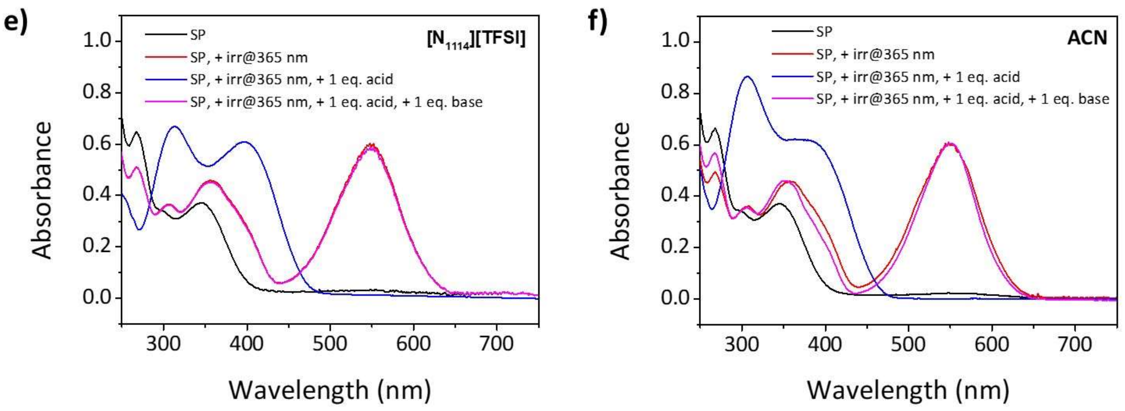

Figure S5. Multistimuli Response of a 0.5 mM solution of NO_2BIPS: 1) acid-base halochromism **a)** in [N$_{1114}$][TFSI] and **b**) acetonitrile; 2) acid-irradiation halophotochromism **c)** in [N$_{1114}$][TFSI] and **d**) acetonitrile; and 3) irradiation-acid-base photohalochromism **e)** in [N$_{1114}$][TFSI] and **f**) acetonitrile.

7. Halochromic cycles in NO_2BIPS@IG membranes

To test the stability of NO_2BIPS@IG membranes to prolonged acid-base treatment, we monitored by UV-vis absorption spectroscopy several consecutive halochromic cycles of **SP**-(*Z*)-**MCH**$^+$ transformation by sequential addition of H_2SO_4 and TBAOH (Figure S6). Degradation effects were observed after 5 of these cycles, which we ascribe to the base-induced Hofmann elimination of the quaternary ammonium cation of the ionic liquid.[5]

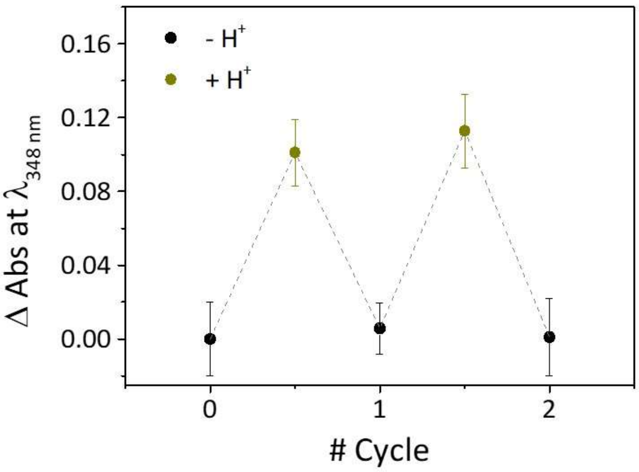

Figure S6. Variation of the absorbance at the spectral maximum of the **SP** isomer in NO_2BIPS@IG upon 2 consecutive cycles of H_2SO_4 (10 mM) and TBAOH (10 mM) addition. Average data is shown for 3 independent measurements in different membranes.

8. Thermochromism of NO_2BIPS in $[N_{1114}][TFSI]$ solution

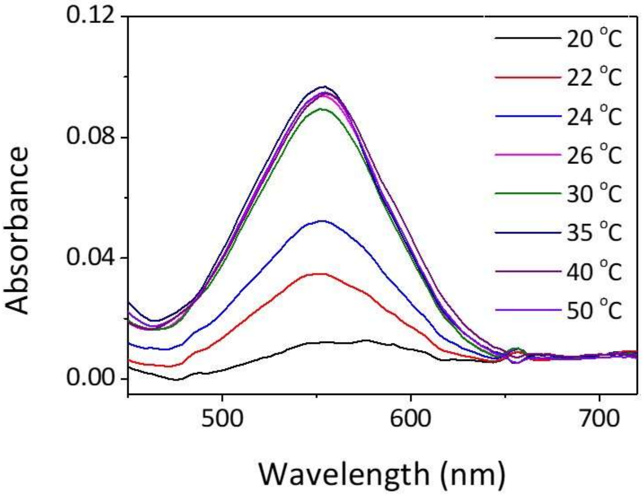

Figure S7. UV-Vis absorption spectra of a 0.5 mM solution of NO_2BIPS in $[N_{1114}][TFSI]$ at different temperatures.

9. Cyclic voltammetry of NO2BIPS@IG membranes

Figure 8 shows the cyclic voltammetry of **SP** and **SP-SP** dimer in IG membranes. The initial NO_2BIPS@IG membrane shows a one electron reversible peak (black line). Whereas, after the oxidative controlled potential electrolysis of the membrane the cyclic voltammogram recorded shows the presence of the reduction peaks corresponding to the dimer. The **SP-SP** species is obtained by an oxidative C-C aryl coupling of two **SP** units. The shape and the peak current values of the reduction peaks of **SP-SP** dimer revealed the presence of two nitro groups, hence two consecutive reversible one-electron reduction electron transfers were observed.

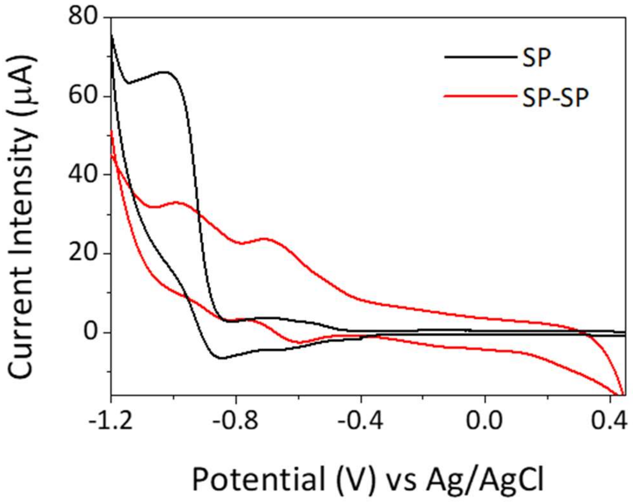

Figure S8. Cyclic voltammogram of NO_2BIPS@IG before (**SP**) and after electrochemically-induced dimerization (**SP-SP**; scan rate: 20 mV s^{-1}).

10. Electrochromic characterization of NO_2BIPS@IG membranes

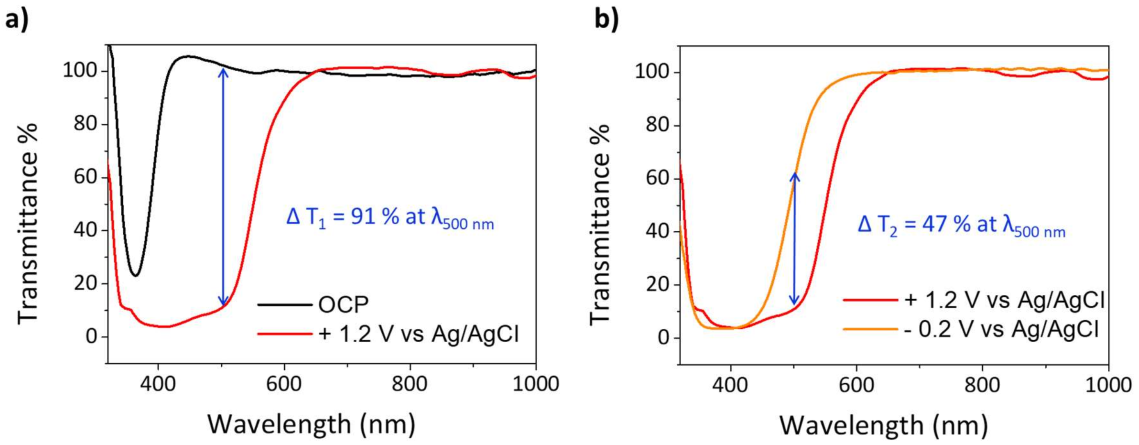

Figure S9. a) Transmittance spectra of NO_2BIPS@IG membranes in the initial **SP** form (OCP, black line) and after applying + 1.2 V (vs Ag/AgCl) for 125 s to form **[SP-SP]**$^{2+}$ (red line). **b)** Change in transmittance spectra when **[SP-SP]**$^{2+}$ is subjected to a potential of + 0.2 V (vs Ag/AgCl) for 60 s to lead to the neutral form **[SP-SP]** (orange line).

11. Fabrication of flexible devices

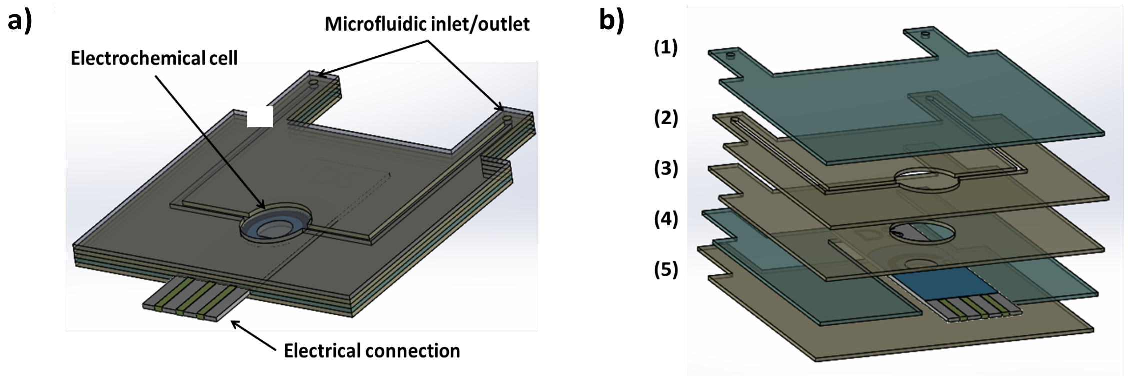

Figure S10. Configuration of flexible devices with an electrochemical cell and microfluidic channels included to apply the different external stimuli. **a)** Device top-view. **b)** Layer-by-layer configuration.

12. Fabrication of rigid microfluidic devices

The microfluidic cell is formed by four PMMA layers (three 500-µm thick and one 3-mm thick) bonded by a 175 µm thick PSA layer, which also hosts the micro-chips used for the electrochromic test (Figure S11). The structure is completed by a 680-µm thick PDMS layer defining the shape and the position of the NO_2BIPS@IG membranes used for the tests. The fluidic interconnections between layers and the corresponding inlets and outlets, and the alignment and insertion of the connectors to contact the electrochemical chips with the measurement instrument were tested prior to the measurements.

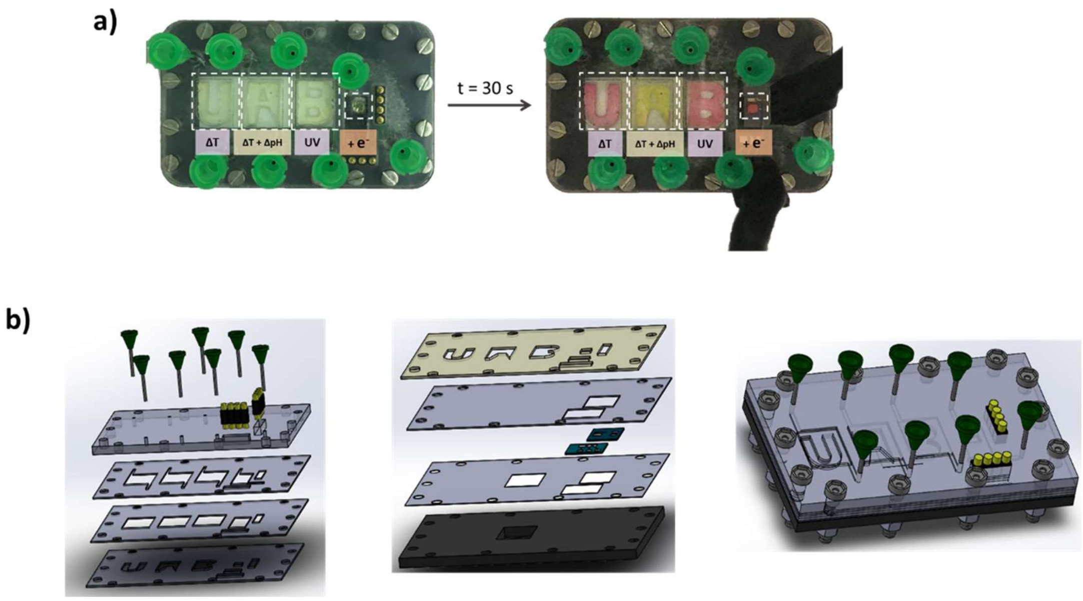

Figure S11. a) Top view of the final prototype structure before (on the left) and after (on the right) the application of the external stimuli for 30 s (ΔT, ΔpH, light and electric potential) on NO_2BIPS@IG membranes shaped into letters and a square. **b)** Scheme of the bottom structure of the microfluidic cell formed by two PMMA layers (3-mm and 380-µm thick) bonded by a 175 µ-m thick PSA layer.

13. References

(1) Görner, H. Photochromism of Nitrospiropyrans: Effects of Structure, Solvent and Temperature. *Phys. Chem. Chem. Phys.* **2001**, *3* (3), 416–423. https://doi.org/10.1039/b007708i.

(2) Lees, A. J. A Photochemical Procedure for Determining Reaction Quantum Efficiencies in Systems with Multicomponent Inner Filter Absorbances. *Anal. Chem.* **1996**, *68* (1), 226–229. https://doi.org/10.1021/ac9507653.

(3) Pimienta, V.; Lavabre, D.; Levy, G.; Samat, A.; Guglielmetti, R.; Micheau, J. C. Kinetic Analysis of Photochromic Systems under Continuous Irradiation. Application to Spiropyrans. *J. Phys. Chem.* **1996**, *100* (11), 4485–4490. https://doi.org/10.1021/jp9531117.

(4) Bandara, H. M. D.; Burdette, S. C. Photoisomerization in Different Classes of Azobenzene. *Chem. Soc. Rev.* **2012**, *41* (5), 1809–1825. https://doi.org/10.1039/c1cs15179g.

(5) Landini, D.; Maia, A.; Rampoldi, A. Stability of Quaternary Onium Salts under Phase-Transfer Conditions in the Presence of Aqueous Alkaline Solutions. *J. Org. Chem.* **1986**, *51* (16), 3187–3191. https://doi.org/10.1021/jo00366a022.
